# Mechanism and optimization of hydrocyclone-based enrichment of calcium and magnesium in fine coal gangue

**DOI:** 10.1371/journal.pone.0339328

**Published:** 2026-01-22

**Authors:** Zhicheng Liu, Hao Pan, Tianqi Song, Zhiwei He

**Affiliations:** 1 China Coal Research Institute Co., Ltd., Xi’an, China; 2 Xi`an University of Science And Technology, Xi’an, China; Cumhuriyet University: Sivas Cumhuriyet Universitesi, TÜRKIYE

## Abstract

Coal gangue, an industrial solid waste generated during coal mining and processing, poses significant environmental challenges due to long-term stockpiling and landfilling. Its comprehensive utilization requires not only decarbonization but also the enrichment of valuable components such as calcium and magnesium through hydrocyclone separation. In this study, the multicomponent occurrence characteristics of coal gangue were examined at the microscale, and the flow-field behavior of hydrocyclones was investigated using computational fluid dynamics (CFD). Based on these insights, a hydrocyclone enrichment system for calcium and magnesium was developed by optimizing cyclone structure and operational parameters. The raw coal gangue exhibited a high ash content (81.66%), mainly composed of Al and Si, with 2.39% Ca and 0.46% Mg. After crushing to 0–1 mm, Ca was enriched in coarse, high-density fractions, while Mg was concentrated in fine, high-density fractions. In the conventional hydrocyclone, increasing feed velocity improved pressure and tangential velocity but caused instability in the locus of zero vertical velocity (LZVV) and air-core morphology, limiting separation accuracy. The bottom-impact hydrocyclone demonstrated superior performance at an impact-tube height of 80 mm and an impact velocity of 5 m/s, achieving improved pressure distribution, higher tangential velocity, and more stable air-core symmetry. Compared with the conventional design, the optimized structure enhanced classification efficiency from 92.47% to 96.13% and increased the Ca content in the underflow to 3.53%. However, Mg separation remained limited under all tested conditions.

## 1. Introduction

Coal gangue is one of the primary solid wastes generated during coal mining and processing, accounting for 15%–20% of total coal output. China’s total stockpile of coal gangue has exceeded five billion tons [1]. Owing to their low calorific value and limited usability, large quantities are stored in open [2], leading to significant land occupation and atmospheric dust pollution. Coal gangue is primarily used for building materials and soil amendment [3], but it has also been developed for high‐value applications like adsorbent materials [4]. All these approaches rely on the separation and enrichment of specific coal gangue constituents to meet performance requirements.

In recent years, with the continuous innovation and improvement of separation technologies, significant progress has been made in the separation of valuable components from coal gangue, with research mainly focusing on gravity separation, flotation, magnetic separation, and intelligent sorting [5]. Gravity separation is an effective technique based on the density differences among materials, in which appropriate separating media and operating conditions are employed to achieve separation between components of different densities and the enrichment of specific fractions. Zhang et al. [6] applied a dense-medium separation method to coal gangue, and the experimental results indicated that appropriately increasing the feed concentration effectively improved the recovery rate of coarse particles (>0.045 mm). Flotation is a beneficiation process that relies on the differences in the hydrophobic and hydrophilic properties of mineral particle surfaces. Bian et al. [7]employed a diesel–Span 80 composite collector to carry out flotation decarbonization of coal gangue, obtaining a clean coal product with an ash content of 19.17% and a combustible recovery of 67.74%. The rapid advancement of artificial intelligence technologies has opened new opportunities for coal gangue separation. Zhang et al. [8] proposed a deep-learning-based non-contact recognition and pneumatic ejection sorting system for the automatic separation of coal gangue, achieving a recognition accuracy of over 97% and a sorting rate exceeding 91%, thereby providing strong technical support for the resource utilization and environmental management of coal gangue. Among these separation methods, hydrocyclone separation, characterized by its simple structure, large processing capacity, high classification precision, and fine cut size, is particularly suitable for the separation of fine coal gangue particles [9]. Moreover, hydrocyclone separation technology has been widely applied in various waste valorization processes, such as metal enrichment from electronic waste, plastic waste sorting, and sludge treatment, as well as in mineral recovery operations, including pre-concentration, tailings discharge, and desliming.

A number of optimization measures to improve fine particles separation efficiency and strengthen the classification process in hydrocyclone separation have been proposed. These include adjusting the hydrocyclone structural parameters and operating conditions. E et al. [10] optimized the traditional inlet structure and found that different inlet types are suitable for different scenarios, with the tangential circle inlet offering a balanced performance in both separation efficiency and energy consumption. In addition, the cylinder-to-cone of the hydrocyclone also significantly affects its separation performance. Studies have shown that a smaller ratio helps stabilize the air core and reduces particle misclassification [11]. Cai et al. [12] used computational fluid dynamics (CFD) to study how different volute wrap angles (0°, 90°, 180°, and 270°) affect the separation efficiency of a dense-medium hydrocyclone, and found that a 180° wrap angle yielded better separation with minimal increase in energy consumption. Honaker R [13] studied the impact of five dual-feed-inlet cross-section shapes on pressure drop and classification efficiency; the rectangular–rectangular inlet produced a tangential velocity far exceeding that of other geometries, whereas circular-square and rectangular-elliptical inlets achieved the lowest pressure drop and highest classification efficiency, respectively. Li et al. [14] have studied the effects of varying the vortex‐finder wall thickness and the insertion of a central solid rod, showing that a thicker vortex‐finder wall effectively reduces short‐circuits flow, whereas the inserted rod mitigates the impact of the air core, and adjusting the rod’s diameter allows fine‐tuning of the cut size. Jiang et al. [15] compared cylindrical, conical, and W-shaped bottom geometries to the conventional cylinder-cone combination, but both simulations and experiments demonstrated that these novel shapes underperformed, thereby confirming the efficacy of the traditional design. Qian [16] studied the effect of increasing the length of the underflow outlet’s straight pipe and observed that, while the tangential and axial velocities, as well as turbulent kinetic energy decreased, thereby enlarging the separation zone, the pressure drop increased. This indicates the existence of an optimal straight pipe length. Jiang et al. [17] further showed that the introduction of an internal conical insert at the underflow outlet can suppress the backflow to a certain extent. In summary, extensive research has been conducted on the optimization of hydrocyclone structural parameters, providing valuable references for practical mineral separation. However, for specific minerals such as coal gangue, studies focusing on enhanced hydrocyclone separation through structural optimization remain limited and warrant further investigation.

Among the operating parameters, feed pressure plays a pivotal role: both separation efficiency and energy consumption scale with inlet pressure, although its influence on separation performance is less pronounced than that of throughput or split ratio [18]. When all other conditions are kept constant and only the inlet pressure is increased, efficiency shows only a slight change while energy consumption rises sharply [19]. The smaller the cyclone’s main diameter, the greater the sensitivity of separation performance to inlet pressure [20]. Consequently, low‐pressure hydrocyclones are preferred to minimize energy usage, with high‐pressure units reserved for cases in which heavy or ultrafine particles cannot be effectively separated under low pressure. Feed solid concentration also critically influences performance; lower concentrations generally enhance separation efficiency [21], but within a sufficiently low range, the impact becomes limited. Therefore, optimizing the feed concentration within an appropriate window is essential to achieve the best separation outcome.

With the rapid advancement in computer technology, CFD, a powerful tool for simulating complex flows, has been widely applied in the study of hydrocyclone. In recent years, numerous CFD studies have examined various hydrocyclone configurations [22,23]. For multiphase rotating flows, mature turbulence models in CFD include the Reynolds stress model (RSM) and large eddy simulation (LES), whereas established multiphase flow models comprise the volume‐of‐fluid (VOF), mixture, and Eulerian approaches [24]. Brennan [25] predicted tangential and axial velocities, as well as radial pressure profiles in a 75 mm hydrocyclone, comparing simulations with laser Doppler velocimetry (LDV) data for both conical and cylindrical sections. Slack [26] simulated the turbulence field in a 205 mm hydrocyclone using RSM and LES, comparing the resulting axial and tangential velocity profiles with LDV measurements. The RSM predictions closely matched the experimental data while demanding lower computational cost and mesh quality than LES. In contrast, LES required finer meshes and smaller time steps, substantially increasing the computational expense. Consequently, RSM is generally preferred for hydrocyclone simulations, and its accuracy has been validated in recent studies. Li et al. [27] adopted LES in their work: they first used VOF to model the gas–liquid two‐phase flow, obtaining velocity and pressure fields, and then introduced solid particles into a two‐fluid model to evaluate separation performance. They found that in a 50 mm hydrocyclone, increasing the vortex finder length caused the tangential velocity and pressure drop to rise and then fall, peaking at 30 mm.

Among the various inorganic components present in coal gangue, Ca and Mg not only govern its separability in hydrocyclone classification but also play a pivotal role in its subsequent resource utilization. Ca and Mg are mainly hosted in calcite and dolomite, respectively, which exhibit pronounced differences in particle size and density after crushing, making them the components most responsive to gravity- and centrifugal-based separation in hydrocyclones. In contrast, Fe-, K-, and Ti-bearing minerals generally occur in lower quantities or lack sufficient physical contrasts to produce meaningful classification behavior, and thus have limited influence on the separation mechanisms considered in this study. Furthermore, CaO and MgO are essential precursors for the formation of calcium silicate hydrate (C–S–H) and magnesium silicate hydrate (M–S–H) phases, and their contents and reactivities directly determine the hydration behavior and mechanical performance of coal gangue–based cementitious materials, alkali-activated binders, and geopolymer products [28,29].

Increasing the Ca–Mg content has been shown to significantly enhance the cementitious activity and strength development of gangue-derived materials, thereby enabling higher substitution ratios in cement clinker, blended cements, and lightweight aggregates and facilitating large-scale utilization [30–32]. Additionally, CaO– and MgO–bearing mineral phases serve as active sites for CO₂ mineralization and for neutralizing acidic media; recent studies have demonstrated that alkaline oxides in coal gangue can be effectively used in mineral carbonation, soil remediation, and solidification, highlighting their potential in carbon reduction and environmental applications [33]. In contrast, although Fe or Ti components also possess certain recovery value, their extraction typically requires strong acid or alkali leaching followed by multistage solid–liquid separation, which may generate secondary tailings and saline or acidic wastewater, thereby imposing additional environmental burdens [34]. Therefore, considering separation feasibility, construction and environmental application value, and the necessity to avoid secondary pollution, Ca and Mg are selected as the primary target components for selective enrichment. This study systematically investigates their occurrence characteristics and separation behavior within hydrocyclone classification.

In this study, the multicomponent microscale occurrence characteristics of a typical coal gangue were first analyzed, and on this basis, CFD was employed to investigate the internal flow field of the hydrocyclone. A gravity-based hydrocyclone enrichment system for Ca–Mg components was then developed. Although numerous studies have improved hydrocyclone performance by optimizing inlet configurations, cylinder-to-cone ratios, vortex finder geometries, and apex structures, these approaches are all based on adjustments to the traditional top-down swirling configuration, and none have introduced an impinging jet at the cyclone bottom to restructure the internal flow field. To address this gap, this study proposes a bottom-impact hydrocyclone design, in which an impinging flow is introduced through an impact outlet at the cyclone base [35]. This configuration establishes a secondary centrifugal acceleration zone, stabilizes the locus of zero vertical velocity (LZVV) and air-core morphology, reduces fine-particle entrainment, loosens the accumulated solids at the cone bottom, and increases internal pressure and tangential velocity—collectively enhancing the centrifugal separation of particles [36–38]. These flow-field regulation mechanisms have not been previously reported. Furthermore, considering the importance of Ca and Mg in coal gangue and the lack of a systematic separation theory and technique for these components, this study integrates microscale occurrence characteristics with flow-field optimization to achieve their targeted enrichment. This provides a novel technical route for the resource utilization and large-scale environmentally friendly disposal of coal gangue.

## 2. Experimental

### 2.1 Test sample

The gangue samples used in this study were collected from the Wangjialing Coal Preparation Plant in Shanxi Province, China. Prior to XRF analysis, all samples were oven-dried at 105°C, ground to <74 μm, and homogenized, followed by pellet pressing using a hydraulic press. XRF measurements were performed using a PANalytical Axios X-ray fluorescence spectrometer. Each sample was analyzed in triplicate, and the standard deviation of elemental concentration was within 3%, ensuring adequate analytical repeatability. X-ray fluorescence (XRF) results are shown in Table 1.

**Table 1:** Elemental composition of the coal gangue sample.

Element	Al/%	Si/%	Fe/%	K/%	Ca/%	Ti/%	Mg/%	Others/%
Content	24.12	41.31	4.93	3.56	2.39	1.59	0.46	21.64

Based on the elemental composition of the sample, Si exhibited the highest content (41.31%), followed by Al (24.12%). The sample also contains Fe, K, Ca, Ti, and Mg. Among these, Ca was present at 2.39% (a minor element), and Mg at 0.46% (a trace element).

**Fig 1:**
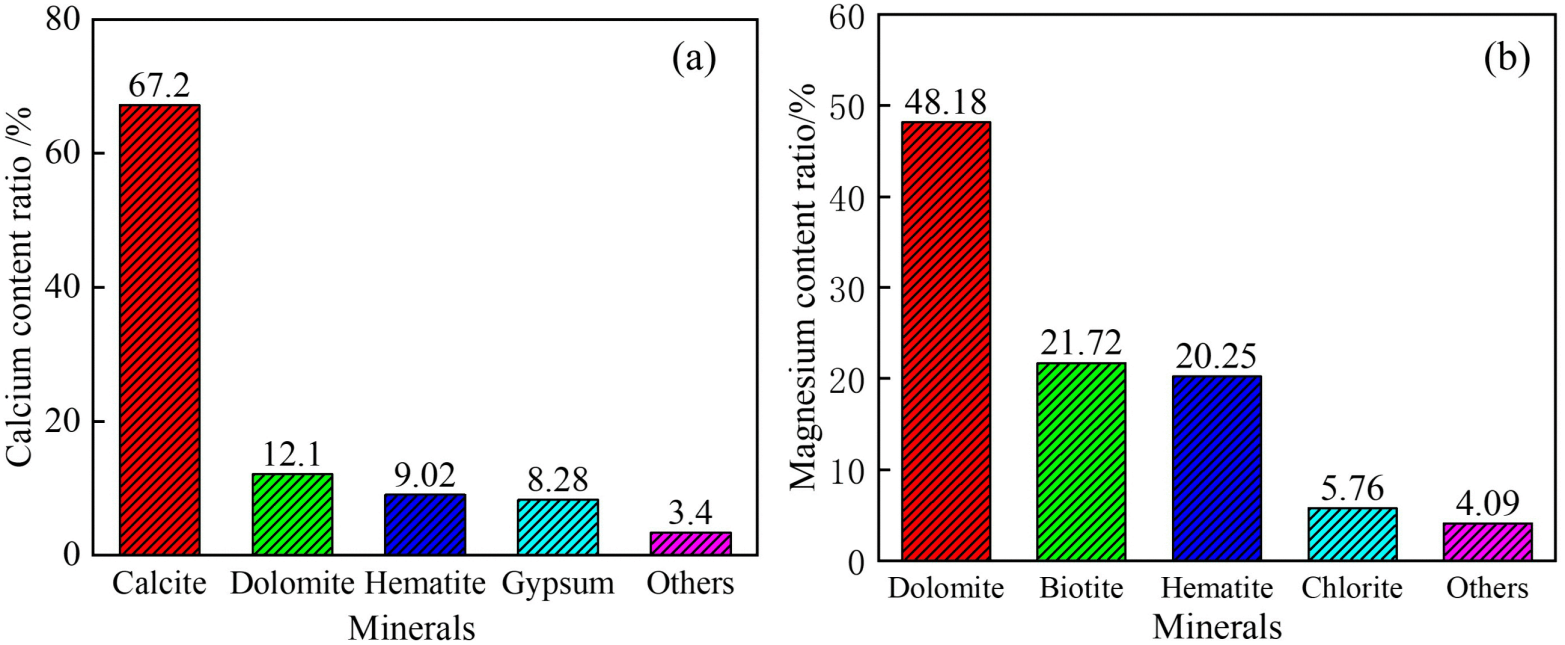
Characteristics of the occurrence of Ca and Mg.

To further analyze the mineral phases present in coal gangue, an MLA (Mineral Liberation Analysis) test was conducted. The occurrence characteristics of calcium and magnesium components are shown in Fig 1. As illustrated, Ca is primarily hosted in calcite, accounting for 67.2% of its total content, while Mg is mainly found in dolomite, representing 48.18%. Moreover, dolomite also contains a significant amount of calcium. Therefore, the separation of calcite and dolomite offers an efficient pathway for the enrichment of calcium and magnesium elements.

In coal gangue, various mineral phases are intergrown and interlocked; therefore, the material must first be crushed and then separated using a hydrocyclone to remove unwanted components and enrich the Ca–Mg fraction for effective recovery. For the preparation of experimental samples in this study, the following procedure was adopted. The bulk sample was homogenized and crushed to <1 mm using a jaw crusher, followed by a roller crusher. The crushed material was then classified by sieving and sink–float separation to obtain coal gangue fractions of different particle sizes and densities. The elemental composition of each size fraction was determined using XRF, and the results are shown in Table 2.

**Table 2:** Grain size distribution and calcium–magnesium content of crushed coal gangue.

Particle Size/mm	Yield/%	Ash/%	Ca/%	Mg/%	Cumulative retained			Cumulative passing		
Yield/%	Ca/%	Mg/%	Yield/%	Ca/%	Mg/%
1 ~ 0.50	27.99	81.35	3.39	0.42	27.99	3.39	0.42	100.00	2.54	0.45
0.50 ~ 0.25	6.56	80.91	3.82	0.45	34.55	3.47	0.43	72.01	2.21	0.46
0.25 ~ 0.125	7.66	81.50	3.01	0.43	42.20	3.39	0.43	65.45	2.05	0.47
0.125 ~ 0.074	12.18	81.86	2.88	0.42	54.39	3.27	0.42	57.80	1.92	0.47
0.074 ~ 0.045	20.64	80.89	1.75	0.44	75.03	2.86	0.43	45.61	1.67	0.49
−0.045	24.97	81.12	1.60	0.52	100.00	2.54	0.45	24.97	1.60	0.52
Total	100	81.24	2.54	0.45						

The results show that after crushing, the coal gangue yield follows a “U-shaped” distribution: the highest yields occur in the −0.074 mm and 0.5–1 mm fractions, while the intermediate-size fractions have lower yields. The ash content varied only slightly across all size fractions and was relatively uniform in the fine fractions, with no significant interfraction differences.

The analysis of calcium content shows that Ca was higher in the coarse fractions, peaking at 3.82% in the 0.5–0.25 mm fraction and reaching a minimum of 1.60% in the −0.045 mm fraction. In terms of cumulative content, the + 0.074 mm fraction contained 3.27% Ca with a corresponding yield of 54.39%, whereas the −0.074 mm fraction contained 1.67% Ca with a yield of 45.61%. Therefore, Ca enrichment could be achieved by collecting the + 0.074 mm coal gangue fraction.

The analysis of magnesium content shows that Mg was relatively low and evenly distributed in the + 0.045 mm fraction but peaked at 0.52% in the 0.045 mm fraction (yield: 24.97%). Cumulative analysis shows that the + 0.045 mm fraction contains 0.43% Mg at a yield of 75.03%, while the −0.045 mm fraction has 0.52% Mg at a yield of 24.97%. Although Mg distribution varied slightly across size fractions, the overall low concentration and limited variability suggest that size classification alone may not be effective for Mg enrichment.

In addition, a sink-float test and Ca/Mg content analysis were performed on coal gangue crushed to 0–1 mm, with results shown in Table 3.

**Table 3:** Density composition and calcium-magnesium content of crushed coal gangue.

Density class (g/cm^3^)	Yield/%	Ash/%	Ca/%	Mg/%	Cumulative float			Cumulative sink		
					Yield/%	Ca/%	Mg/%	Yield/%	Ca/%	Mg/%
﹣1.6	2.08	11.63	1.44	0.02	2.08	1.44	0.02	100.00	2.43	0.41
1.6 ~ 1.7	2.19	29.00	1.15	0.14	4.27	1.29	0.08	97.92	2.46	0.41
1.7 ~ 1.8	2.58	42.54	1.44	0.12	6.86	1.35	0.10	95.73	2.49	0.42
1.8 ~ 2.0	7.84	47.99	1.73	0.11	14.69	1.55	0.10	93.14	2.51	0.43
2.0 ~ 2.2	8.96	68.53	3.27	0.17	23.66	2.20	0.13	85.31	2.59	0.46
2.2 ~ 2.4	13.94	77.33	3.79	0.35	37.60	2.79	0.21	76.34	2.51	0.49
+2.4	62.40	91.98	2.22	0.52	100.00	2.43	0.41	62.40	2.22	0.52
Total	100.00	80.06	2.43	0.41						

The results indicate that the yield of coal gangue in the low-density fractions is relatively low, with the −1.8 g/cm^3^ fraction yielding only 4.77%. As the density increased, the yield increased steadily, reaching 62.40% in the + 2.4 g/cm^3^ fraction. The ash content decreased with increasing density, suggesting that low-density gangue was enriched in residual carbon, whereas high-density gangue contained less carbon.

The analysis of calcium content shows that Ca also varies with fraction density, increasing as density increases, which indicates that Ca is predominantly hosted in higher-density fractions. In the float fraction (<2.0 g/cm^3^), cumulative Ca content was only 1.55% at a mass yield of 14.69%; in the sink fraction (>2.0 g/cm^3^), cumulative Ca increased significantly to 2.59% at a mass yield of 85.31%. These findings demonstrate that density-based physical separation can effectively enrich Ca, although the enrichment effect is less pronounced than that achieved by the size classification.

The analysis of calcium content shows that Mg exhibited a distribution characteristic similar to that of Ca, with progressive enrichment with increasing density. Mg content peaks at 0.52% in the + 2.4 g/cm^3^ fraction. In the low-density floats (<2.2 g/cm^3^), cumulative Mg content is 0.13% at a mass yield of 23.66%, whereas in the high-density sinks (>2.2 g/cm^3^), cumulative Mg increases to 0.49% at a yield of 76.34%. Therefore, density separation is effective not only for Ca enrichment but also for Mg enrichment.

### 2.2 Experimental and numerical simulation methods

#### 2.2.1. Numerical simulation methods.

**Fig 2:**
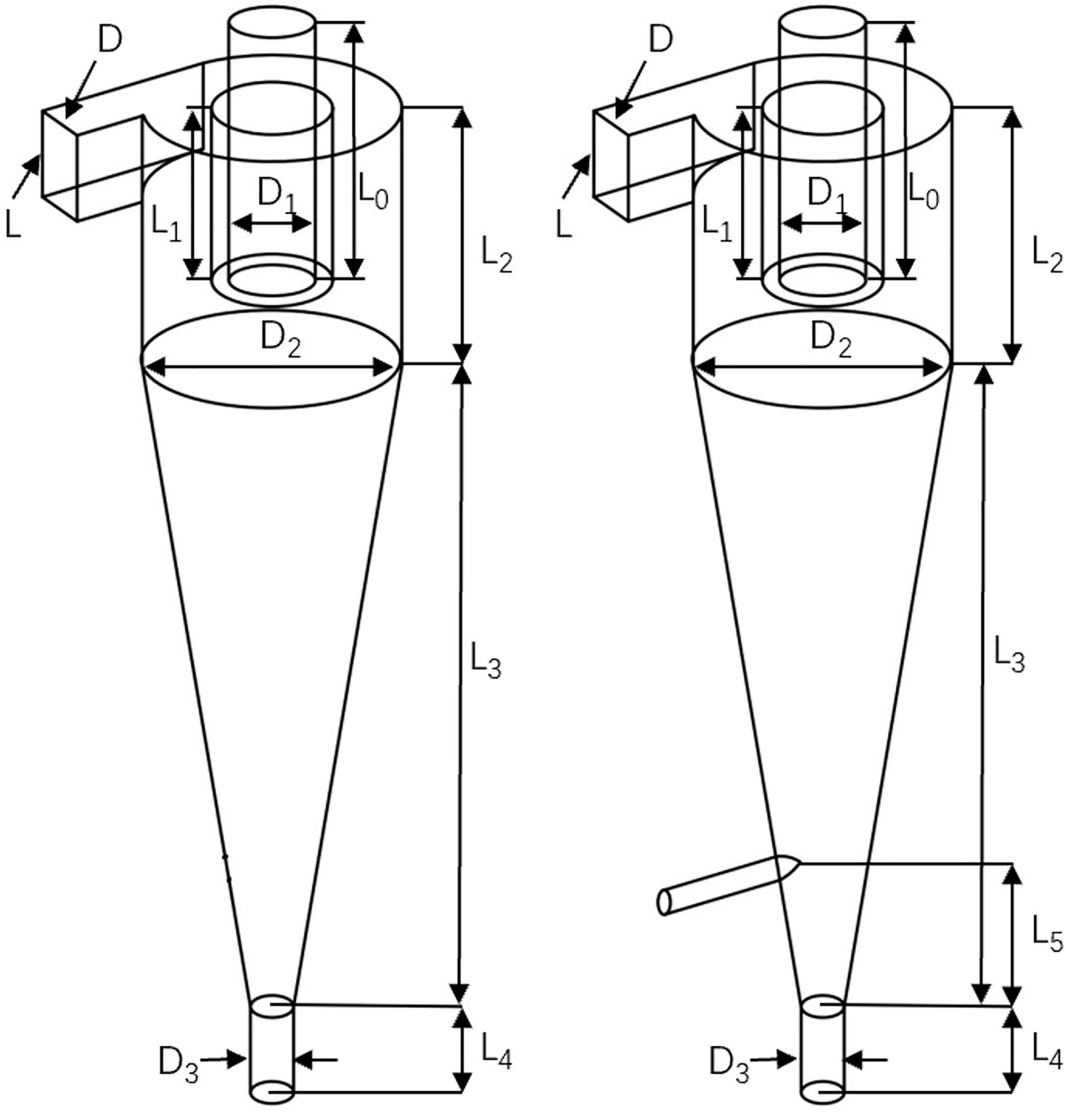
Structural schematic diagram of conventional cyclone and bottom-impact cyclone.

The main structural parameters of the bottom-impact hydrocyclone are shown in Table 4, and the schematic diagrams of a conventional hydrocyclone and bottom-impact hydrocyclone are shown in Fig 2. By incorporating an impact-port structure at the base of the cone, the impact cyclonic separator introduced an impinging flow that stabilized the overall flow field, reduced recirculation zones and turbulence near the underflow outlet, and lowered the probability of fine particles being entrained into the underflow, thereby improving both separation accuracy and efficiency. The added impact port also exerted an additional impulse on the particles at the base of the cone, increasing the internal pressure of the hydrocyclone. This pressure increase accelerated the fluid flow inside the cyclone, which in turn increased the centrifugal force acting on the particles, providing a secondary centrifugal acceleration that further aided in the classification and separation of coal gangue particles. In addition, the injected impinging flow loosened the particles at the base of the cone, further strengthening the separation performance.

**Table 4:** Main structural parameters of the conventional cyclone and bottom-impact cyclone.

Hydrocyclone structural features	Dimension/mm
Feed Inlet Dimensions (D × L)	20 × 25
Overflow Pipe Diameter (D_1_)	25
Overflow Pipe Length (L_0_)	75
Overflow Pipe Insertion Depth (L_1_)	50
Cylindrical Section Diameter (D_2_)	75
Cylindrical Section Height (L_2_)	75
Conical Section Height (L_3_)	186
Underflow Outlet Height (L_4_)	25
Underflow Outlet Diameter (D_3_)	12.5
Overflow Pipe Wall Thickness (D_5_)	5
Impact Pipe Height (L_5_)	40,60,80,100,120

**Fig 3:**
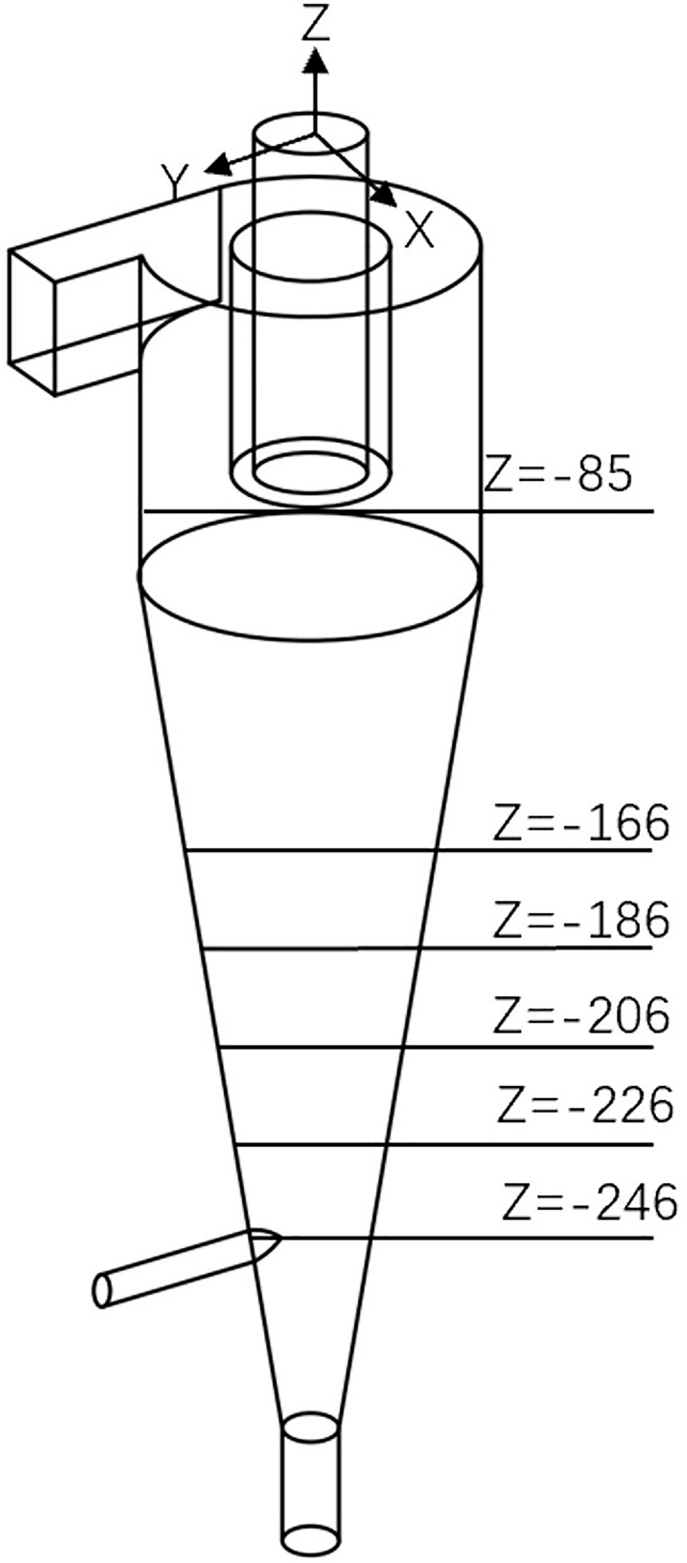
Characteristic structural diagram of bottom-impact cyclone.

As shown in Fig 3, in order to comprehensively examine and analyze the internal pressure field, velocity fields and air-core morphology of the bottom-impact hydrocyclone. Contour plots of the pressure distribution, tangential velocity, axial velocity, and air core were generated at several cross-sections (X = 0, Z = −85 mm, −166 mm, −186 mm, −206 mm, −226 mm, and −246 mm).

This study used UG 3D modeling software to construct the internal flow‐field geometries of both the conventional hydrocyclone and the bottom‐impact hydrocyclone. The completed geometric models were then imported into ANSYS ICEM for mesh generation of the fluid domain. An unstructured tetrahedral mesh was employed to ensure adequate geometric adaptability and flow‐field resolution. To further improve the accuracy in regions with strong velocity and pressure gradients, local mesh refinement was applied at the feed inlet, bottom‐impact inlet, air‐core region, vortex‐finder outlet, and the lower cone–underflow zone. In addition, near‐wall mesh elements were refined to maintain y⁺ < 30, ensuring compatibility with the standard wall‐function approach. The finalized mesh was subsequently imported into ANSYS Fluent for numerical simulation.

To ensure numerical accuracy, a grid‐independence study was performed on an impact‐type hydrocyclone mesh. Using a model with the impact‐port length L_5_ = 80 mm as an example, six mesh schemes with progressively increasing cell counts (82,797, 184,095, 253,159, 367,807, 503,046, and 627,021) were generated. The tangential velocity at the cross sections X = 0, Z = −85 mm was chosen as the key metric for assessing the influence of mesh density on the simulation results.

**Fig 4:**
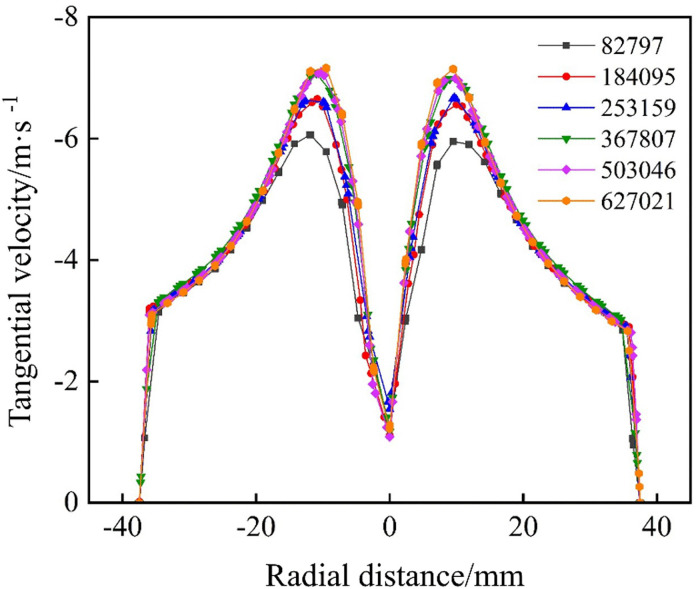
Grid independence verification.

Fig 4 depicts the tangential velocity distribution at this point for each mesh. After reaching a mesh size of 367,807 cells, further refinement had no significant impact on the velocity profile, indicating that the solution was mesh‐independent. Accordingly, a 367,807-cell mesh was selected for all the subsequent simulations.

Three multiphase models commonly used in Fluent: Mixture, Eulerian, and VOF. Among these, the VOF model is preferred for capturing the free interface between the air core and the liquid phase. Therefore, the VOF model was used to simulate the internal air core dynamics in the hydrocyclone.

Due to high‐speed fluid rotation within a confined space, the flow becomes highly turbulent and complex. Therefore, an appropriate turbulence model is required. Common choices include the RSM, LES, k–ε model, and k–ω model. The standard k–ε model tends to produce excessive turbulent viscosity and unrealistic tangential velocities, making it unsuitable for the accurate description of rotational flow. Although both RSM and LES can resolve the turbulence in a hydrocyclone, LES demands more computational resources. RSM strikes a balance with lower mesh and hardware requirements and shorter runtimes, making it the selected turbulence model.

In terms of the boundary conditions, the inlet velocity was initially set to 1.0, 1.5, 2.0, 2.5, and 3.0 m/s to analyze its effect on the internal flow field of the hydrocyclone and to determine the optimal inlet velocity using the experimental data. The hydrocyclone’s structural parameters were optimized based on the optimal inlet velocity. After determining the optimal impact‐port height, the impact velocities of 3, 4, 5, 6, and 7 m/s were studied to see how they affect classification and separation performance.

The inlet boundary condition was defined as a velocity inlet, and the turbulence characteristics were determined using the Turbulence Intensity and Hydraulic Diameter Method. The hydraulic diameter of the feed inlet was set to 25 mm, and the flow field was modeled as a two-phase system, with the water-phase volume fraction set to 1 and the air-phase volume fraction set to 0. The impact port was also defined as a velocity inlet, where the turbulence parameters were specified using the same method. The hydraulic diameter of the impact port was 8 mm, the volume fraction of the clean-water phase was set to 1, and both the air-phase velocity and volume fraction were set to 0. The overflow and underflow outlets were both defined as pressure outlets, with the relative pressure set to 0 (connected to the atmosphere). The hydraulic diameters of the overflow and underflow outlets were 25 mm and 12.5 mm, respectively. Because the high-speed rotation of the internal fluid in the hydrocyclone generates a low-pressure core, which induces air recirculation, the air recirculation coefficients at the overflow and underflow outlets were both set to 1, while the water recirculation coefficients were set to 0.

During numerical initialization, the internal flow domain of the hydrocyclone was initialized as the air phase, and the air-phase volume fraction was set to 1 at t = 0, in accordance with the actual operating conditions. To improve computational accuracy, no-slip wall conditions were applied to all wall boundaries, and standard wall functions were used to handle near-wall turbulence. In terms of solver settings, the SIMPLE algorithm was adopted for pressure–velocity coupling, while the QUICK scheme was used for the spatial discretization of Turbulent Kinetic Energy, Turbulent Dissipation Rate, and Reynolds Stresses. The convergence residuals for all parameters were set to 10 ⁻ ⁴, and other parameters were kept at their default values. The numerical solution was performed using a two-stage procedure. First, a steady-state simulation was conducted to obtain an initially converged flow field, which effectively accelerated the overall computation and improved numerical stability. The converged steady-state field was then employed as the initial condition for the subsequent transient simulation, enabling the capture of the time-dependent evolution of the air-core structure, pressure fluctuations, and LZVV dynamics. This staged steady–transient strategy ensured both computational efficiency and an accurate representation of the intrinsically unsteady flow characteristics within the hydrocyclone. After completing the computation, the simulation results were imported into Tecplot software for post-processing, enabling an in-depth analysis of the internal flow-field characteristics of the conventional hydrocyclone under different feed velocities, as well as those of the bottom-impact hydrocyclone under varying impact-pipe heights and impact velocities.

#### 2.2.2. Hydrocyclone separation test method.

**Fig 5:**
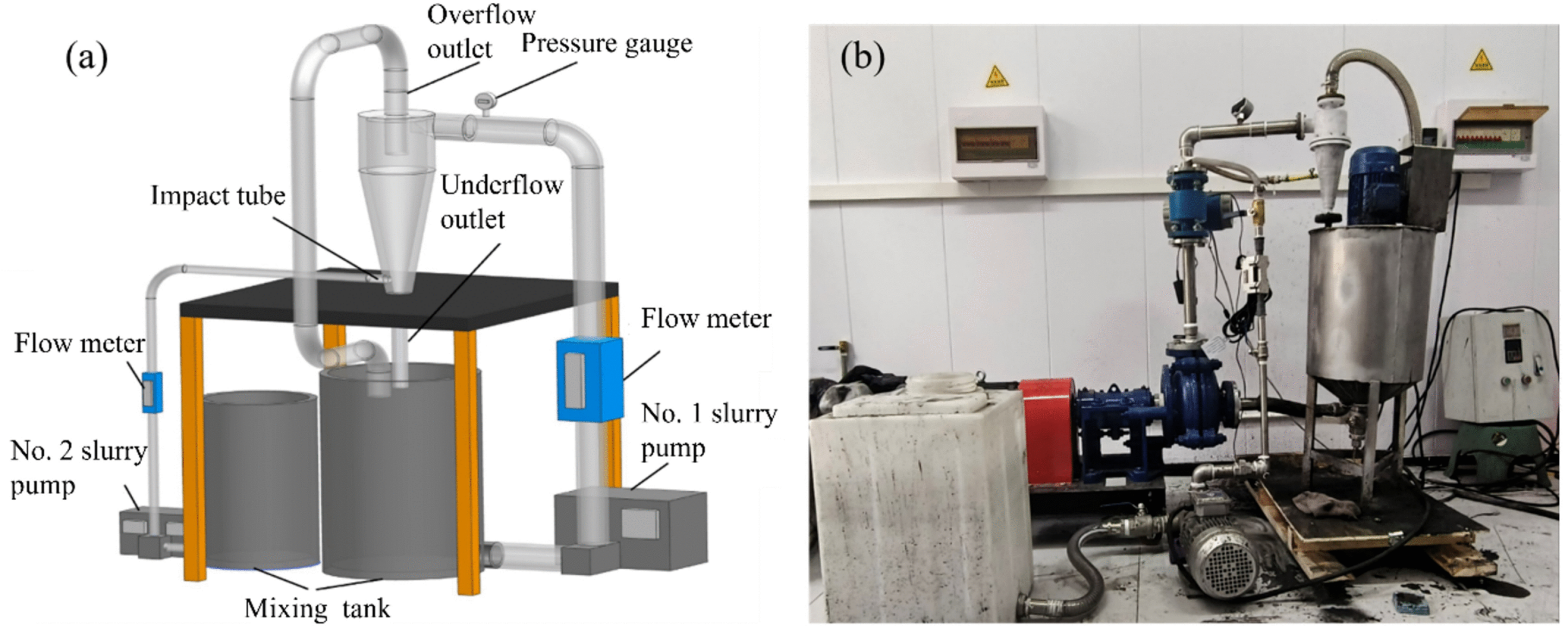
Theoretical and actual diagrams of the cyclone separation enrichment system for calcium and magnesium components. (a) Theoretical diagram; (b) Actual diagram.

A 3D-printed hydrocyclone was used to construct a laboratory-scale hydrocyclone system for the Ca–Mg fraction separation and enrichment. The schematic and physical setup of the system are shown in Fig 5.

The apparatus comprised two subsystems: a hydrocyclone separation system and an impact system. The hydrocyclone separation system included a mixing tank, slurry pump, flow meter, and pressure gauge. The mixing tank thoroughly agitated the coal‐gangue slurry before entering the hydrocyclone, ensuring a uniform feed. The slurry pump served as the core power unit, delivering sufficient kinetic energy to transport the slurry into the cyclone at the required velocity and pressure. The flow meter continuously monitored the slurry flow rate, while the pressure gauge provided real‐time readings of the inlet pressure to the hydrocyclone, which comprised a slurry‐conditioning tank, a slurry pump, a flow meter, and control valves. Introducing clean water tangentially at the cyclone’s base loosened settled coal gangue particles, thereby preventing their accumulation. This impingement also contributed to a secondary classification step, which improved overall grading and separation efficiency of the hydrocyclone.

In the experiment, the crushed and dried coal gangue sample was subsampled using the quartering method, and a 2.05 kg portion was placed into the mixing tank of the hydrocyclone separation system along with 15 L of water. The mixer was operated at 20 Hz for 10 min to produce slurry with a mass concentration of 12%. Following homogenization, the optimal inlet velocity of the conventional hydrocyclone was determined. The first slurry pump was started, forming flow rates of 1.8, 2.7, 3.6, 4.5, and 5.4 m^3^/h, corresponding to inlet velocities of 1.0, 1.5, 2.0, 2.5, and 3.0 m/s. Prior to sampling, the overflow and underflow flow rates as well as the inlet pressure were continuously monitored. Steady-state conditions were considered to be achieved when the flow rates fluctuated within ±2%, the pressure variation was within ±0.5 kPa, and the air-core shape observed through the inspection window remained stable without periodic oscillation. Typically, it requires 3–5 minutes to reach full stabilization. Prior to sampling, the overflow and underflow flow rates as well as the inlet pressure were continuously monitored. Steady-state conditions were considered to be achieved when the flow rates fluctuated within ±2%, the pressure variation was within ±0.5 kPa, and the air-core shape observed through the inspection window remained stable without periodic oscillation. Once the flow stabilized, overflow and underflow fractions were collected for analysis to determine the optimal inlet velocity. Subsequently, the effects of the impact-port height and impact velocity were investigated. Hydrocyclones fitted with different impact-port heights were mounted on the system. The first pump was operated for 10 min until its flow rate stabilized, and the second pump and impact-port valve were then opened to introduce clean water tangentially into the cyclone base at flow rates of 0.54, 0.72, 0.90, 1.09, and 1.27 m^3^/h, corresponding to impact velocities of 3, 4, 5, 6, and 7 m/s. After stabilization, the overflow and underflow products were collected and dried. Then a 200-mesh standard screen was used to sieve the sample. The underflow fine-entrainment ratio was defined as the mass percentage of the −200-mesh fraction in the underflow, and the overflow coarse-carryover ratio referred to the mass percentage of the + 200-mesh fraction in the overflow. Hancock’s classification efficiency (*E*) was used to evaluate the overall separation performance of the hydrocyclone:


$$E=\frac{\left(\alpha -\theta \right)\left(\beta -\alpha \right)}{\alpha \left(\beta -\theta \right)\left(1-\alpha \right)}\times 100\%$$ (1)

where *α* denotes the calculated feed yield of the hydrocyclone, %; *β* is the calculated overflow yield of the hydrocyclone,%; and *θ* represents the calculated particle–size yield in the hydrocyclone underflow, %.

The ash content of the collected overflow and underflow products was determined to evaluate the performance of the hydrocyclone in density-based separation. The related formulae are as follows:


$$Ad_{u,d}=\frac{m_{1}}{m_{2}}\times 100\%$$ (2)

Additionally, XRF analyses were conducted to quantify their calcium and magnesium contents of the overflow and underflow products, thereby evaluating the effectiveness of the hydrocyclone in separating and enriching these components.

For all operating conditions, each hydrocyclone separation test was repeated at least three times, and the average values of product indicators were calculated based on valid data to minimize errors and ensure repeatability. For each sample, ash content and XRF analyses were conducted using parallel sample testing to minimize potential instrument-related errors as much as possible. Error analysis indicated that the testing errors for all measurements were within 3%, and the errors for key indicators in the hydrocyclone separation experiments—including product yield, misplacement rate, ash content, and grade—were all within 8%, demonstrating a high level of experimental reliability. Furthermore, no specific permits were required for the fieldwork conducted in this study because the study site is publicly accessible and no protected species were involved.

## 3. Results and discussions

### 3.1 Design of the hydrocyclone separation process and analysis of Flow‐Field characteristics

#### 3.1.1. Analysis of flow field characteristics of conventional cyclones with different feed velocities.

**Fig 6:**
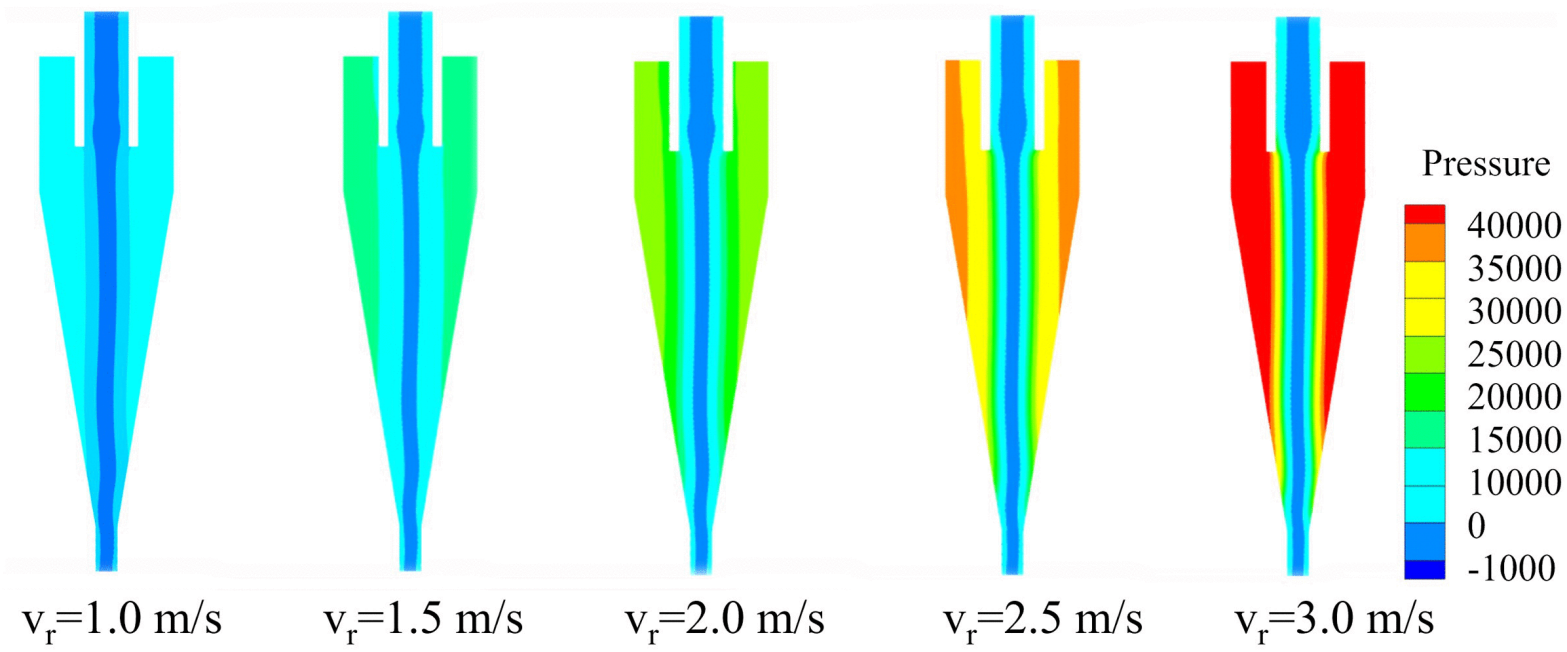
Pressure distribution of the conventional hydrocyclone under different feed velocities.

The pressure distribution of the conventional hydrocyclone at various inlet velocities (v_r_) is shown in Fig 6. As shown, the internal pressure field of the hydrocyclone was axially symmetric and decreased radially outward. Near the central axis, the gauge pressure dropped below zero, resulting in the formation of an air core. As the inlet velocity increased, the static pressure along the cyclone wall increased markedly, indicating that higher feed velocity intensified internal fluid motion and imposed greater radial loads on the wall during rotation. This increase in radial static pressure steepened the pressure gradient, subjecting particles to stronger centripetal buoyant forces. Consequently, fine particles more readily migrated into the inner vortex and were discharged through the overflow. Moreover, the pressure in the cylindrical section exceeded that in the conical section, demonstrating that as the cyclone radius diminished, a greater portion of pressure energy was converted into kinetic energy. These higher pressures and larger pressure drops were conducive to enhanced particle separation.

**Fig 7:**
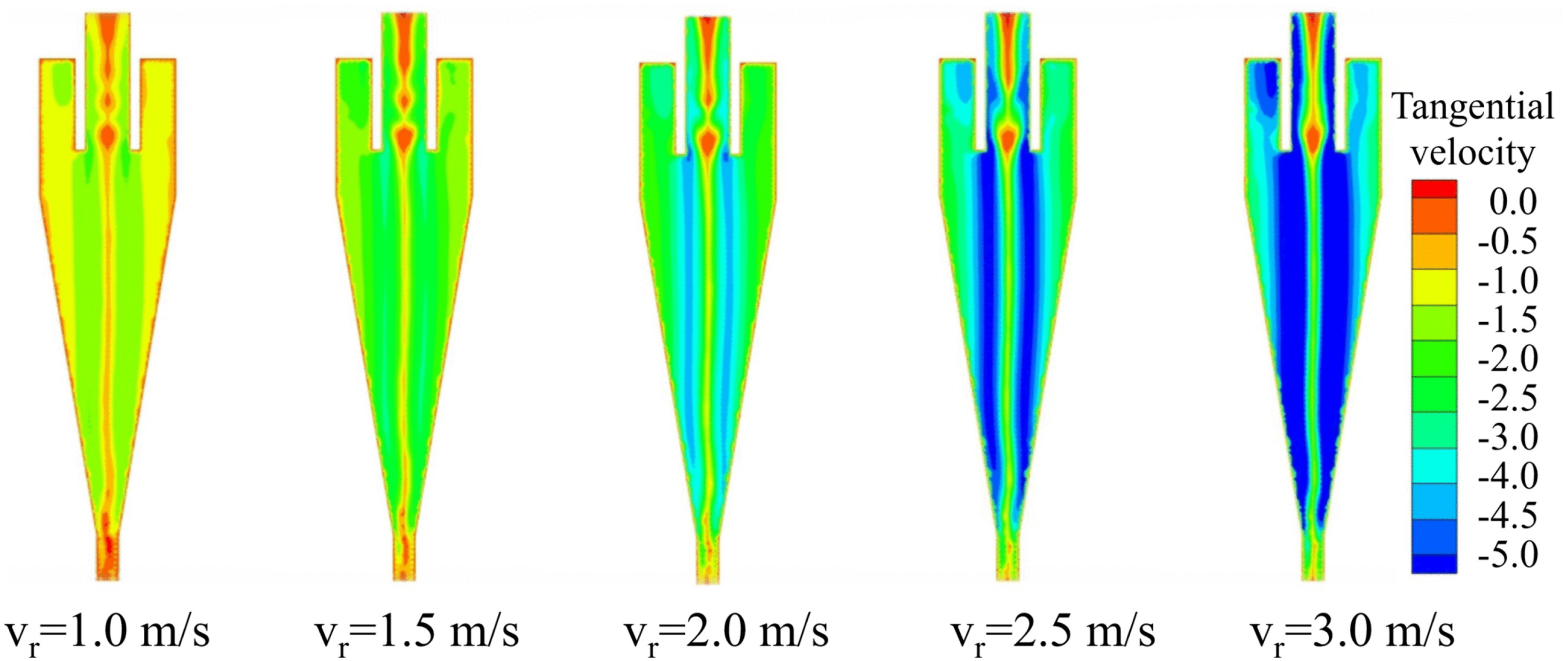
Tangential velocity distribution of the conventional hydrocyclone under different feed velocities.

Among the three velocity components in the three-dimensional flow field, the tangential velocity is the highest, and the centrifugal force acting on particles is predominantly determined by this component. Therefore, the tangential velocity plays a critical role in the classification and separation performance of the hydrocyclone. Fig 7 presents the tangential velocity distribution of the conventional hydrocyclone under different inlet velocities. The results show an axisymmetric pattern in which the tangential velocity increases from the wall toward the core and then decreases near the center. As the inlet velocity increases, the overall tangential velocity within the cyclone also rises, a trend consistent with the observed changes in the pressure field. A higher inlet velocity increases the kinetic energy of the fluid, thereby enhancing the tangential velocity. The strengthened tangential velocity intensifies the centrifugal forces acting on particles, enabling denser or larger particles to migrate more effectively toward the cyclone periphery and thus improving the classification efficiency.

**Fig 8:**
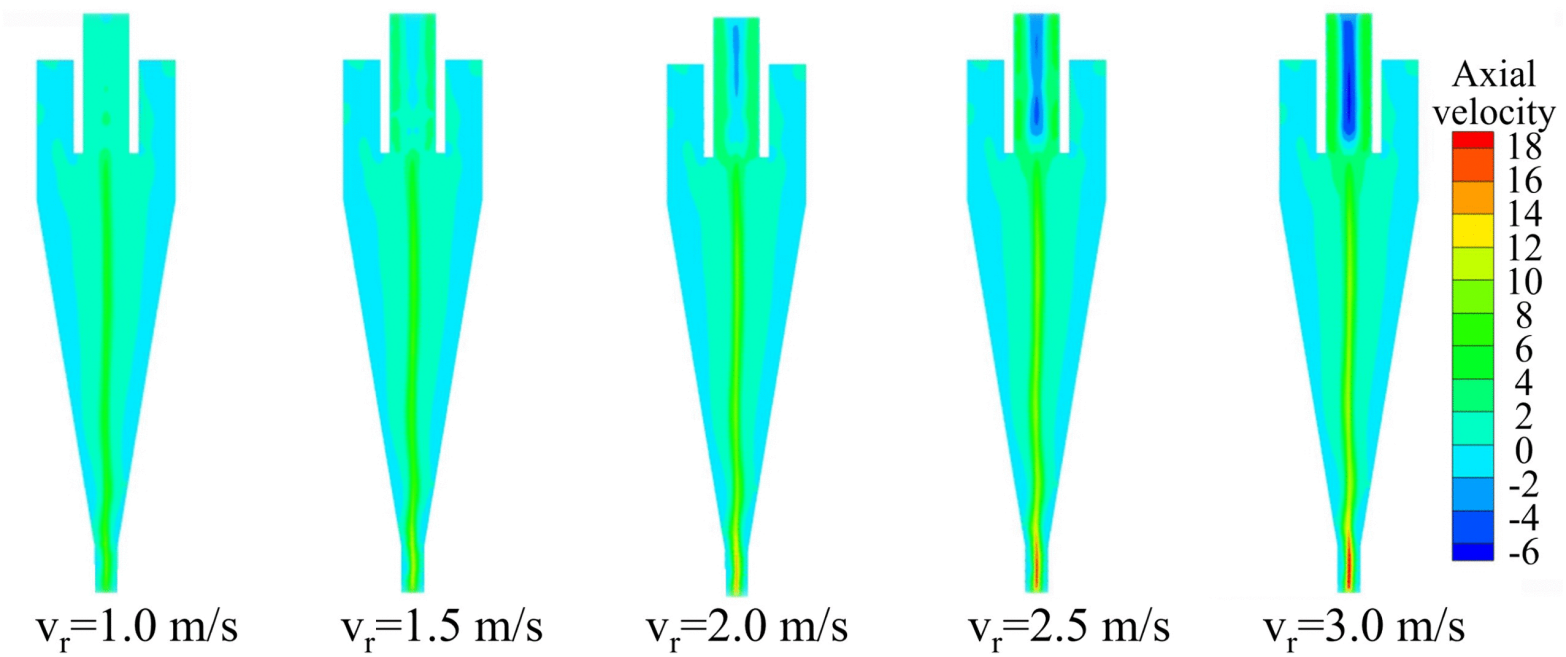
Axial velocity distribution of the conventional hydrocyclone under different feed velocities.

The axial velocity represents the bidirectional flow within the hydrocyclone and is a key velocity component. This not only influences the split ratio but also determines the particle residence time, thereby affecting the separation performance. The axial velocity distribution of the conventional hydrocyclone at different inlet velocities is shown in Fig 8. Within the cyclone, the axial velocity was divided into two flow regions: the outer vortex and the inner vortex. The locations where axial velocity changed sign formed zero‐velocity points that connected to create the zero‐velocity envelope surface, which is the locus of zero vertical velocity (LZVV). This surface delineated the interface between the inner and outer vortices. Fluid inside the LZVV moved upward and exited via the overflow outlet, whereas fluid outside the LZVV traveled downward along the cyclone wall and discharged through the underflow outlet. The shape and position of the LZVV directly affected the separation zone between coarse and fine particles, thus governing the overall separation efficiency. In Fig 8, LZVV is indicated by the boundary between the green and blue regions. Although the axial velocity distributions were approximately symmetric, fluctuations in the LZVV shape at the base of the conical section induced alternating changes in the separation zone, undermining separation stability and impairing classification performance. Changes in inlet velocity had only a minor effect on the axial velocity of the outer vortex, and the overall LZVV geometry remained largely constant across the tested feed velocities.

**Fig 9:**
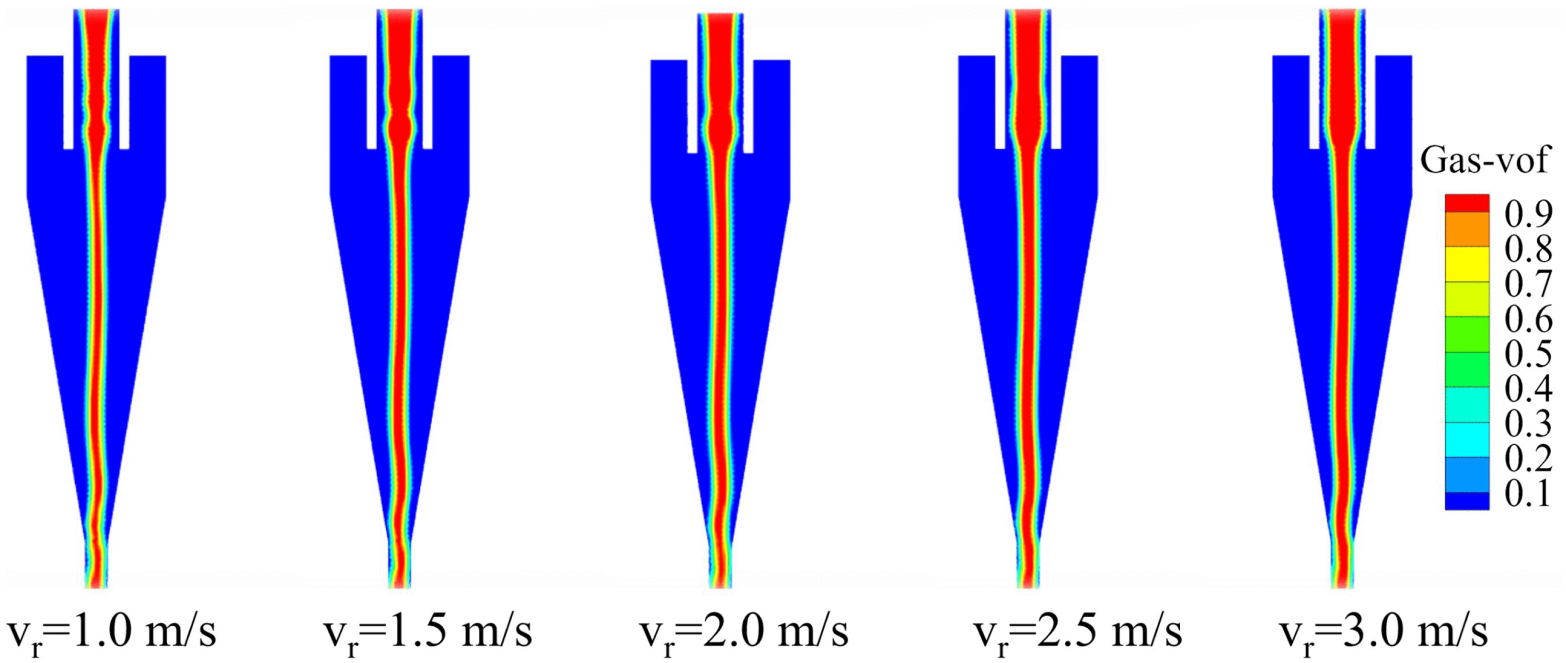
Air column cloud map under different inlet velocities in the conventional hydrocyclone.

The air core is a distinctive phenomenon that develops in the hydrocyclone under the action of internal–external pressure differentials. No particle separation occurs within the air core, but its shape and stability are key indicators of the overall stability of the gas–liquid two-phase flow field. The gas distribution of the conventional hydrocyclone at different inlet velocities is shown in Fig 9. At all tested feed velocities, the air core fluctuated at the base of the conical section of the cyclone, which was detrimental to flow‐field stability. The air core diameter reached its maximum at the bottom of the overflow pipe—corresponding to the well-known “throat bulge”—a feature attributable to multiple convergence points at that location. As the inlet velocity increased, the air core within the overflow pipe progressively enlarged, while the section below the overflow pipe expanded initially before stabilizing. The results indicate that at low inlet velocities (1.0–1.5 m/s), slight oscillations occur at the bottom of the air core, with an interface fluctuation amplitude of approximately 10%. As the inlet velocity increases to 2.0–2.5 m/s, the air core becomes more continuous with smoother axial variation, and the fluctuation amplitude decreases to around 6%. When the inlet velocity reaches 3.0 m/s, the air core is overall the most stable, although minor local disturbances can still be observed near the cone bottom. Overall, increasing the inlet velocity enhances the continuity and axial symmetry of the air core, but it also leads to greater air entrainment, causing the air core to expand more readily in regions of strong swirling motion.

#### 3.1.2. Analysis of flow field characteristics under different impact pipe heights of the bottom impact hydrocyclone.

**Fig 10:**
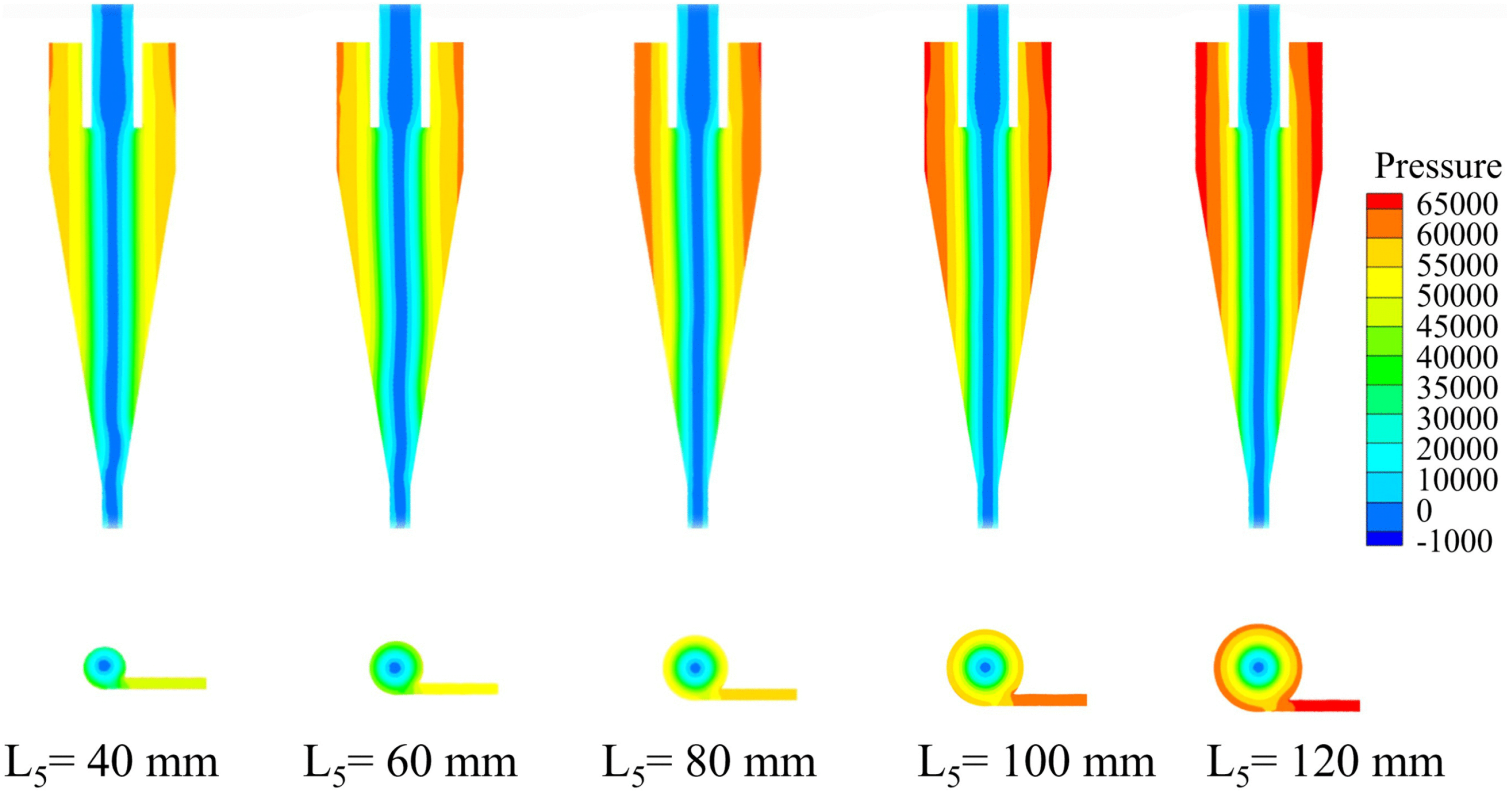
Pressure distribution of bottom-impact hydrocyclone at different impact pipe heights.

**Fig 11:**
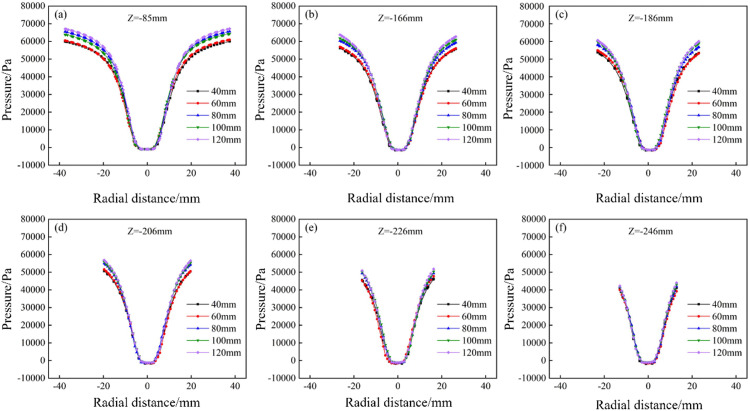
Pressure distribution at X = 0 and different sectional heights under different impact pipe heights in the bottom-impact hydrocyclone. (a) Z = −85 mm, (b) Z = −166 mm, (c) Z = −186 mm, (d) Z = −206 mm, (e) Z = −226 mm, and (f) Z = −246 mm.

Based on the numerical simulation results of the conventional hydrocyclone, the inlet velocity was fixed at 3.0 m/s for the numerical simulations of the bottom-impact hydrocyclone with varying impact pipe heights. The pressure distribution at different impact pipe heights (L_5_) is shown in Fig 10. The pressure profiles along the X = 0 plane at the Z = −85, −166, −186, −206, −226, and −246 mm sections are shown in Fig 11. As shown, the internal pressure clearly decreased with increasing radial position and even fell below zero near the cyclone axis. As the impact pipe height increased, the overall pressure increased—most noticeably at the cyclone wall—indicating that a taller impact pipe intensified the internal fluid motion and subjected the wall to higher radial static pressures during rotation. As shown in Fig 11, all sectional pressure curves exhibit a “V” shape characteristic. With increasing pipe height, the pressure at the wall increased, whereas the pressure inside the air core remained essentially constant, resulting in a larger overall pressure drop. This behavior suggests that a greater impact pipe height increases the centripetal buoyant force on the particles, making it easier for fine particles to migrate toward the core and be discharged through the overflow outlet.

**Fig 12:**
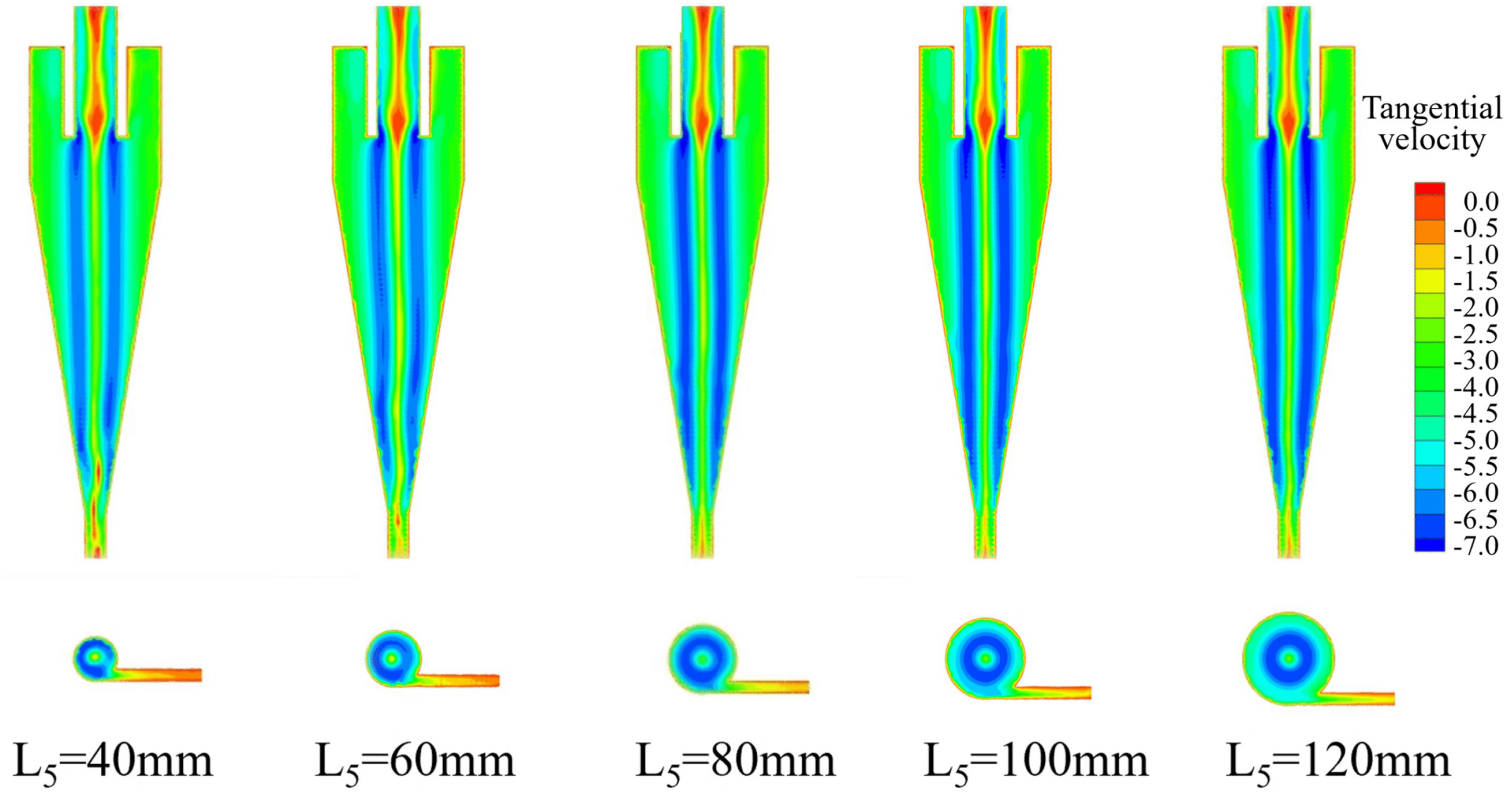
Tangential velocity distribution of the bottom-impact hydrocyclone at different impact pipe heights.

**Fig 13:**
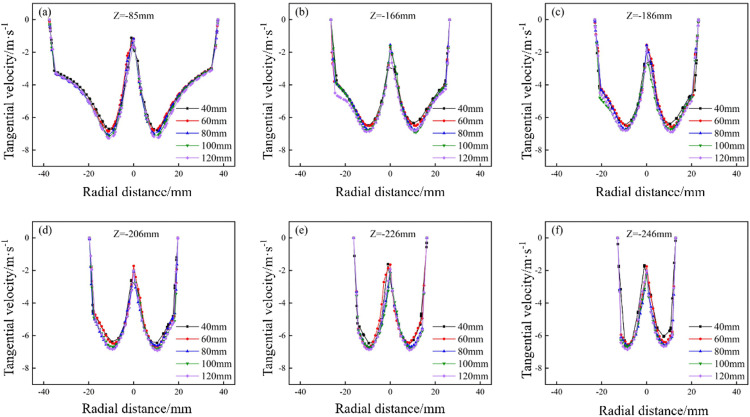
Tangential velocity distribution at X = 0 and different sectional heights under different impact heights in the bottom-impact hydrocyclone. (a) Z = −85 mm, (b) Z = −166 mm, (c) Z = −186 mm, (d) Z = −206 mm, (e) Z = −226 mm, (f) Z = −246 mm.

The tangential velocity distribution of the bottom‐impact hydrocyclone at varying impact pipe heights is shown in Fig 12. The tangential velocity profiles on the X = 0 plane at Z = −85, −166, −186, −206, −226, and −246 mm are shown in Fig 13.

As shown in Fig 12, increasing the impact pipe height had a significant impact on the internal flow field, as tangential velocity increased with axial symmetry. Fig 13 shows a “double‐peak” distribution, where velocity increases from the wall to the core, then decreases near the axis, resulting in a low‐velocity central region. As the impact pipe height increased, the overall magnitude of the tangential velocity also increased, implying a stronger centrifugal force field that enhanced the outward migration of particles. The centrifugal force directly governs the classification and separation efficiency, where a higher force allows larger or denser particles to escape the inner vortex and exit with the underflow. Increasing the pipe height increased the separation efficiency by driving the particles toward the wall.

However, at very low impact pipe heights, tangential velocity symmetry deteriorated, reducing flow stability and separation performance (most pronounced in Fig 12(f)). Similarly, excessively high pipe heights also disrupted symmetry and induced anomalous flow structures, which adversely affected the cyclone’s classification and separation efficiency.

**Fig 14:**
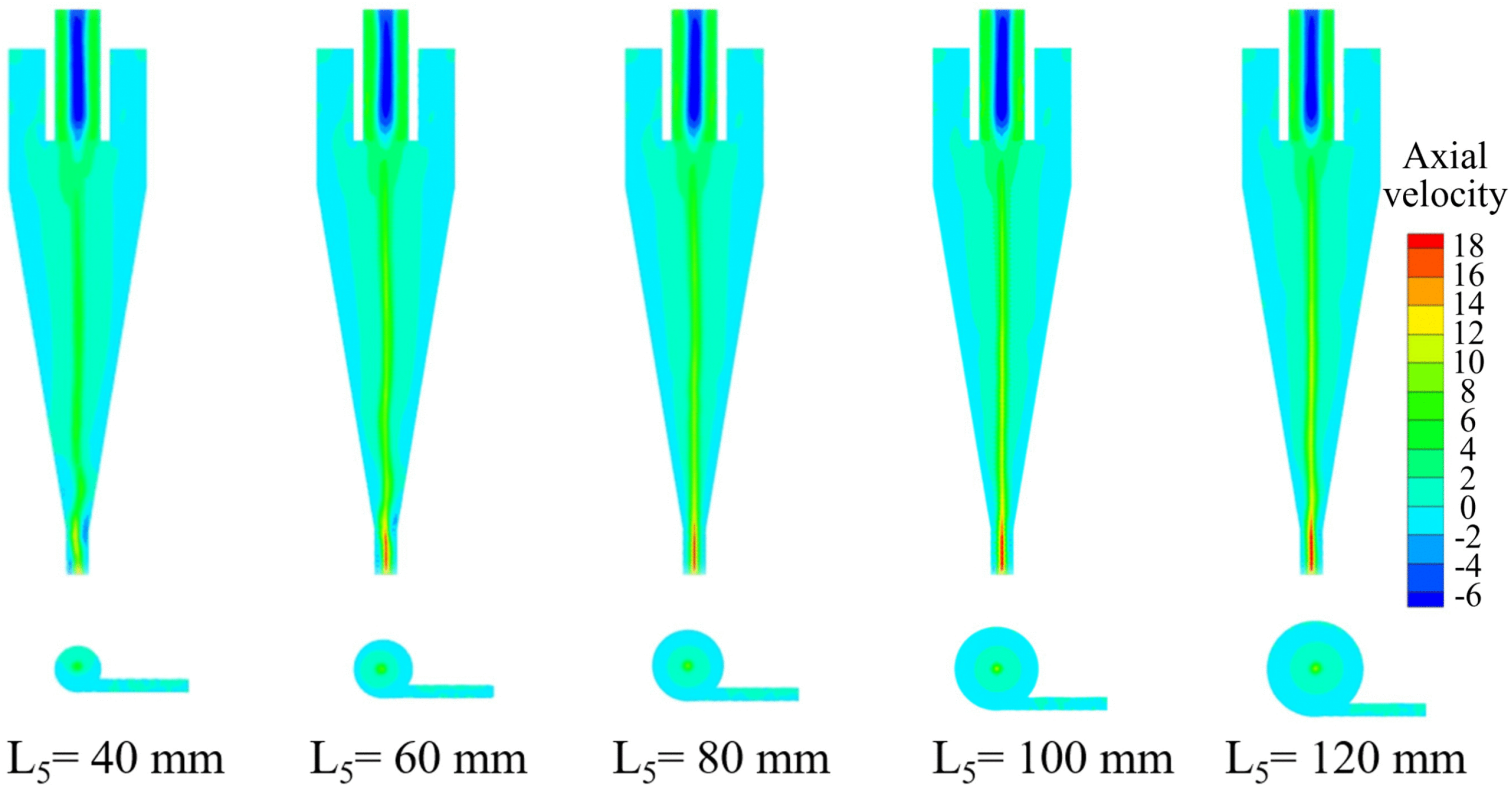
Axial velocity distribution of the bottom-impact hydrocyclone under different impact pipe heights.

**Fig 15:**
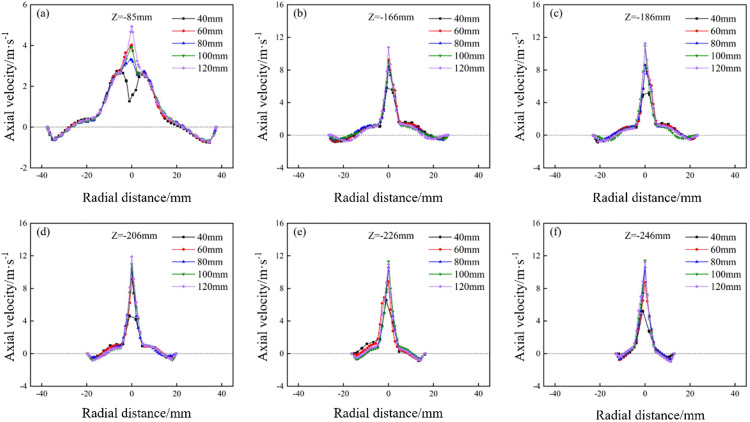
Axial velocity profiles of the bottom-impact hydrocyclone at X = 0 and different section heights. (a) Z = −85 mm, (b) Z = −166 mm, (c) Z = −186 mm, (d) Z = −206 mm, (e) Z = −226 mm, (f) Z = −246 mm.

The axial velocity distribution of the bottom‐impact hydrocyclone at different impact pipe heights is shown in Fig 14. The axial velocity profiles on the X = 0 plane at Z = −85, −166, −186, −206, −226 and −246 mm are shown in Fig 15.

The distribution in Fig 14 indicated that the axial velocity field was largely symmetric, reflecting a reasonably stable flow. The impact pipe height caused a pronounced effect on the shape of the LZVV. Ideally, LZVV should form a regular cylindrical shape that is similar to the internal flow characteristic. At low impact pipe heights, the LZVV displayed significant fluctuations and irregularities at the base, leading to unstable slurry flow in the separation zone and reduced classification performance. As the pipe height increased (particularly beyond 80 mm), the LZVV gradually showed a more regular and symmetric shape, indicating that the internal flow became more stable, thereby improving the separation efficiency. However, further increases in pipe height abruptly re-emerged LZVV fluctuations at the cyclone base region, potentially resulting in anomalous flow structures that impaired particle classification.

As shown in Fig 15, the axial velocity magnitude increased from the wall toward the axis, peaked at the center, and then decreased, with a significant change in flow direction. Low impact pipe heights resulted in an asymmetric and unstable flow field, making classification difficult. When the pipe height reached 80 mm or more, the symmetry of the axial velocity profiles improved, indicating that taller impact pipes enhanced the stability of the internal flow field and supported a more consistent separation performance.

**Fig 16:**
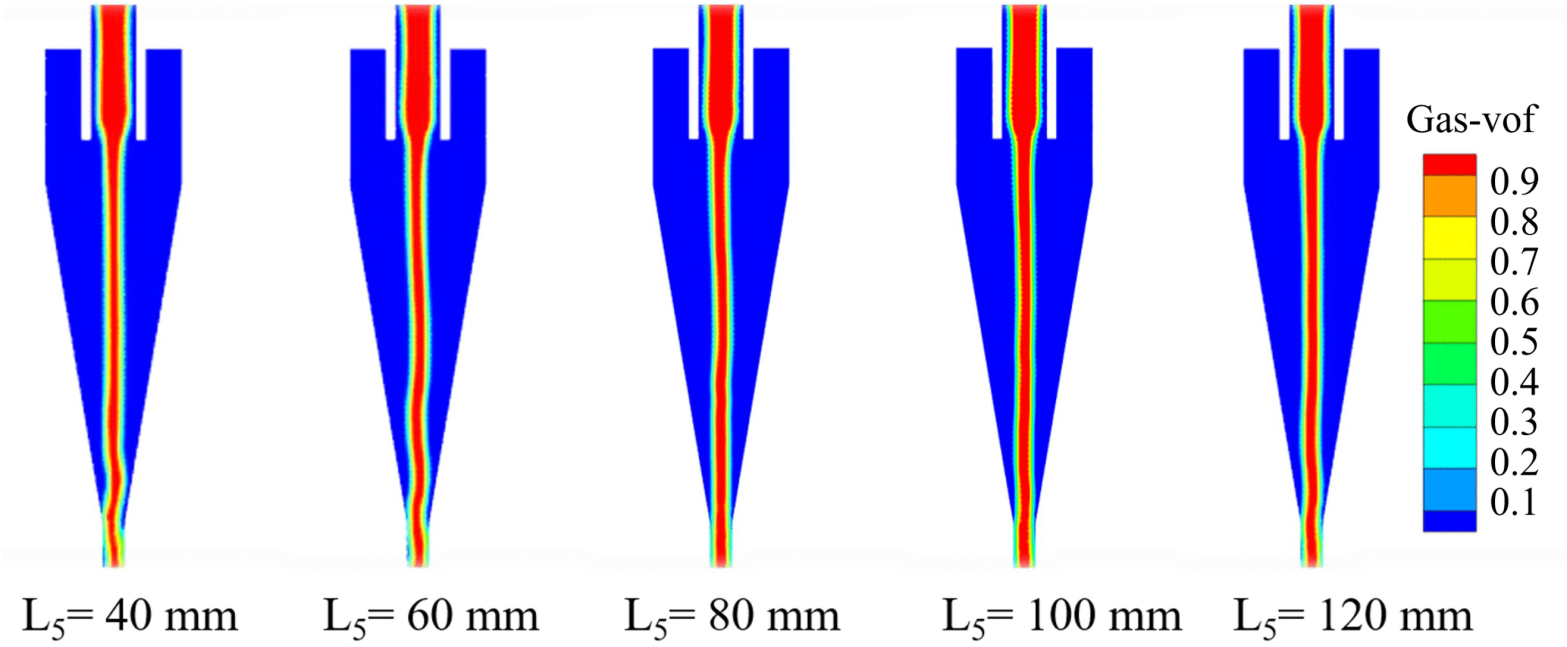
The air column in the bottom-impact cyclone under different impact pipe heights.

The air‐core contours of the bottom‐impact hydrocyclone at different impact pipe heights are shown in Fig 16. The air core is a distinctive flow feature of the cyclone separation process, and its stability reflects the overall stability of the flow field.

Fig 16 shows how the height of the impact pipe significantly affects the formation and stability of the internal air core. At low pipe heights, the air core was highly irregular and exhibited pronounced fluctuations at its base, leading to an unstable flow field that was detrimental to separation performance. With increasing pipe height, the air‐core shape became progressively more regular, and its axial distribution became more uniform, indicating enhanced flow‐field stability and improved classification and separation. However, when the pipe height was further increased to 120 mm, the air core fluctuations intensified again, potentially impairing the separation efficiency. For the bottom-impact hydrocyclone, the impact-pipe height L_5_ was varied from 40 to 120 mm. The revised text now specifies that at L_5_ = 80 mm the air core becomes nearly axisymmetric with a uniform axial extent, whereas at lower heights (40–60 mm) the air core exhibits strong fluctuations and at L_5_ = 120 mm the fluctuations intensify again, indicating an optimum structural window around L_5_ ≈ 80 mm for maintaining air-core stability.

#### 3.1.3. Analysis of flow field characteristics under different impact velocities of bottom impact cyclones.

**Fig 17:**
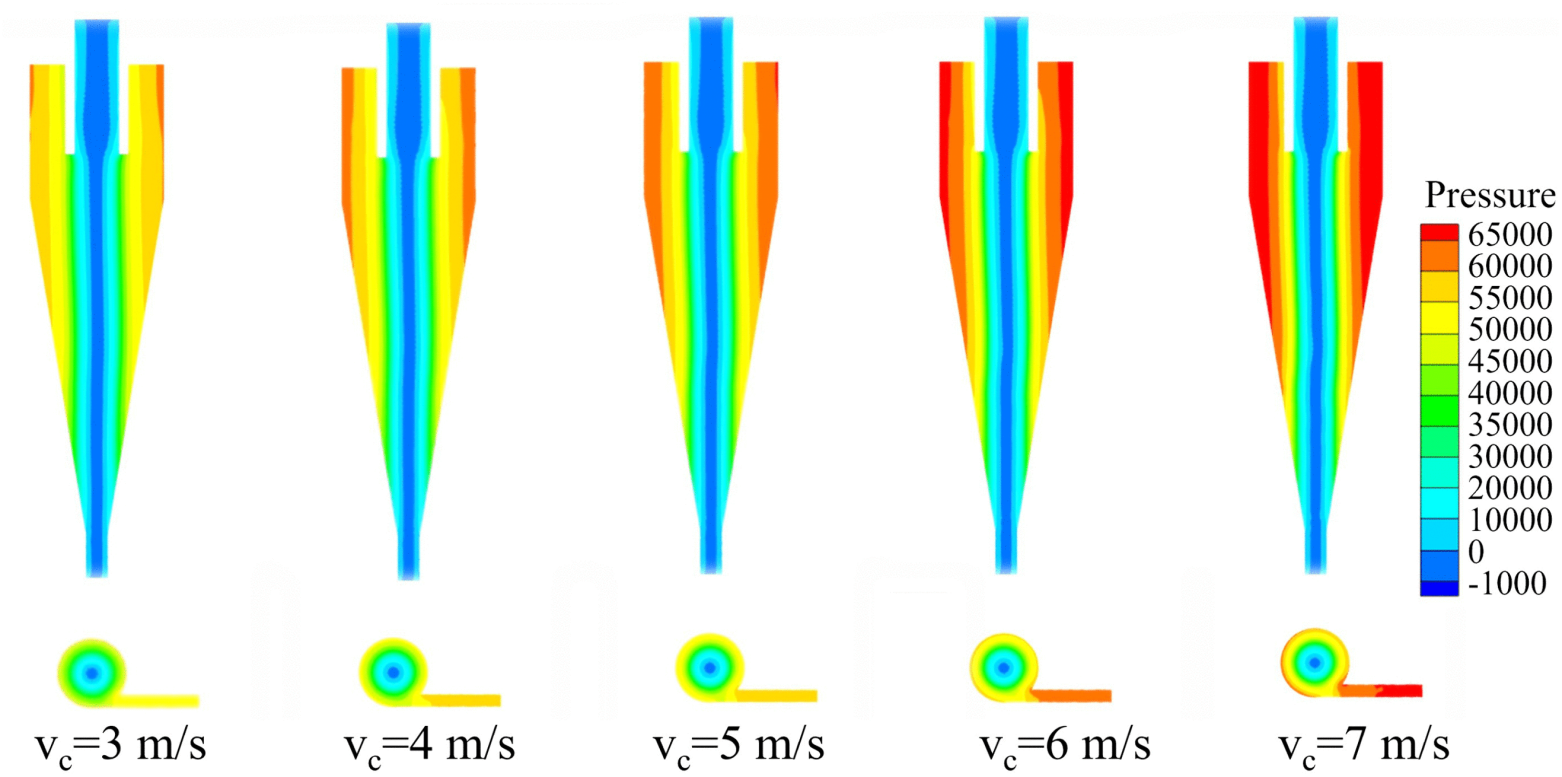
Pressure distribution of the bottom-impact cyclone under different impact velocities.

**Fig 18:**
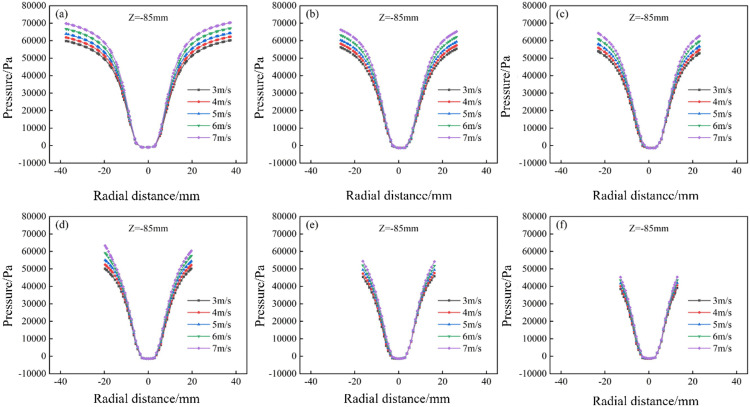
Pressure distribution diagrams at the X = 0 plane and different height sections under various impact velocities in the bottom-impact cyclone. (a) Z = −85 mm, (b) Z = −166 mm, (c) Z = −186 mm, (d) Z = −206 mm, (e) Z = −226 mm, and (f) Z = −246 mm.

The pressure distribution contours of the bottom‐impact hydrocyclone at different impact velocities (v_c_) are shown in Fig 17. The pressure profiles along the X = 0 plane at Z = −85, −166, −186, −206, −226, and −246 mm are shown in Fig 18.

As shown, the internal pressure exhibited a clear radial decrease, reaching a minimum (sub‐atmospheric) at the core due to rotational flow and then rose symmetrically toward the wall. As the impact velocity increased, the overall pressure level also increased, indicating that higher velocities increased radial static pressure on the wall and converted more pressure energy into kinetic energy—both of which favored particle separation.

As shown in Fig 18, each sectional pressure exhibited a “V” shape characteristic. The lowest pressure occurred at the core, and the pressure steadily increased with radial distance. With increased impact velocity, the entire curve shifted upward, especially near the wall. This elevation of the pressure profile increased the radial pressure gradient, thereby increasing the centripetal buoyant force on the particles. The steeper slope of the pressure distribution at higher velocities further amplified this gradient, helping finer or lower‐density particles overcome centrifugal repulsion and move inward into the inner vortex. Consequently, both the underflow fineness and overflow coarseness rates decreased, leading to an improvement in overall classification efficiency.

**Fig 19:**
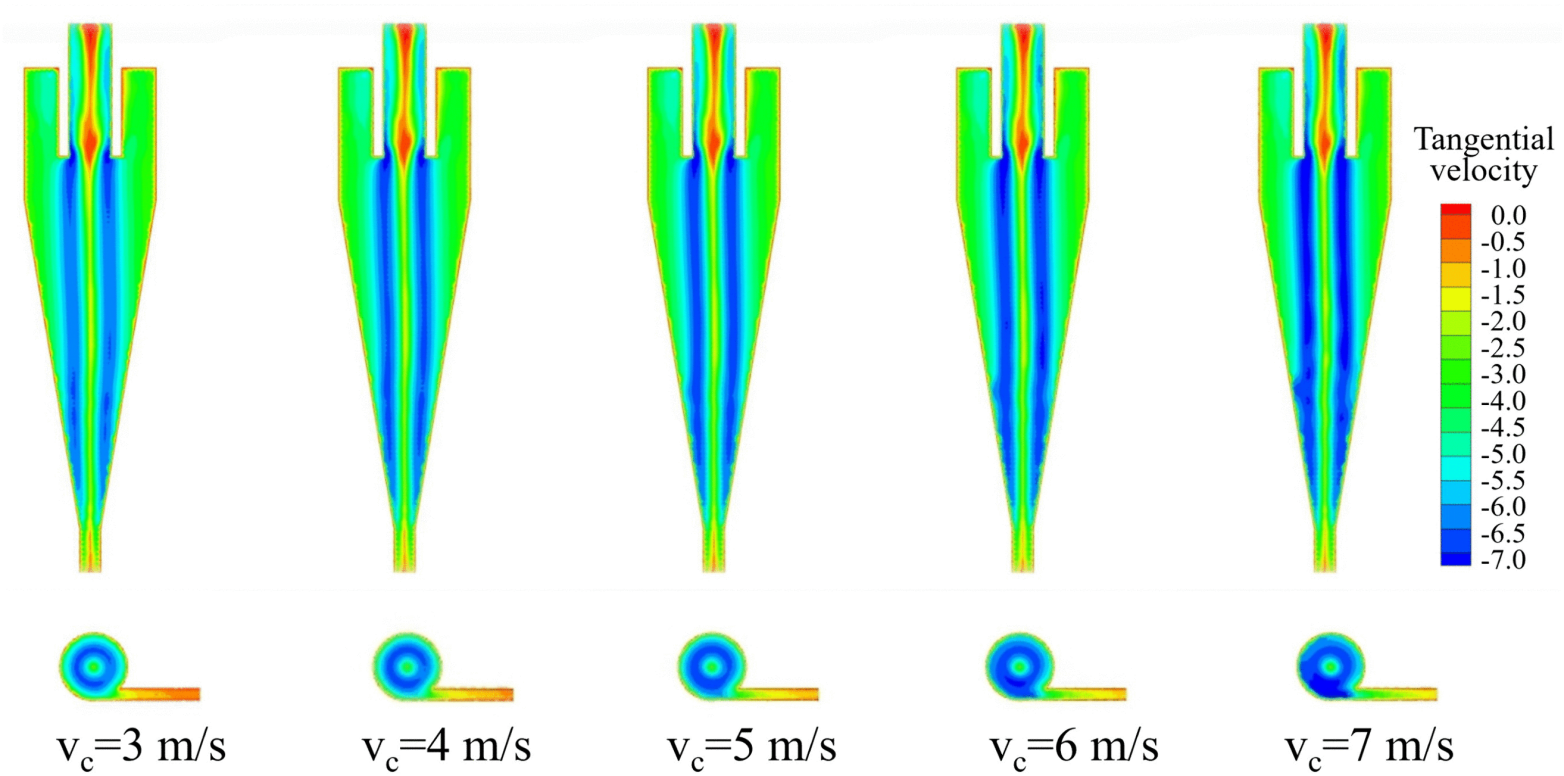
Tangential velocity distribution under different impact velocities in the bottom-impact hydrocyclone.

**Fig 20:**
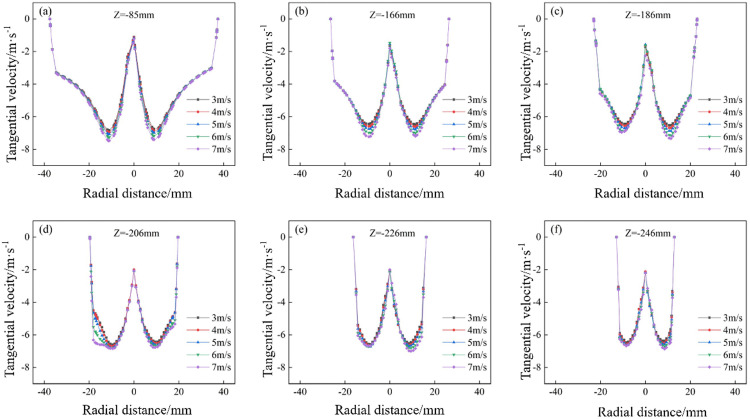
Pressure distribution diagrams at the X = 0 plane and different height sections under various impact velocities in the bottom-impact cyclone. (a) Z = −85 mm, (b) Z = −166 mm, (c) Z = −186 mm, (d) Z = −206 mm, (e) Z = −226 mm, (f) Z = −246 mm.

The tangential velocity distribution of the bottom‐impact hydrocyclone at different impact velocities is shown in Fig 19. The tangential velocity profiles on the X = 0 plane at Z = −85, −166, −186, −206, −226, and −246 mm are shown in Fig 20. From Fig 19, the tangential velocity was axially symmetric and increased with impact velocity, indicating that higher velocities strengthened the centrifugal field and thus the centrifugal force acting on particles. This demonstrates how impact velocity affects the internal flow field and the forces governing particle motion. Fig 19 reveals a “W”–shaped characteristic profile at each section. The tangential velocity rose from the wall toward the core, peaked, then declined to form a low‐velocity zone near the axis. As the impact velocity increased, the entire profile shifted upward, reflecting an intensified centrifugal force field that improved the ability of the particles to migrate from the inner vortex to the outer vortex. In the −85 mm and −166 mm sections (Figs 20a and 20b), the profiles remained symmetric across all velocities. In the lower sections (Figs 20c–20f), symmetry was maintained at modest velocities but degraded at the highest velocities—most notably in Fig 20d—despite the overall velocity increase. As shown in Fig 19, this asymmetry at high velocities coincided with pronounced tangential velocity fluctuations near the cyclone base, which can undermine the flow stability and adversely affect the separation performance.

**Fig 21:**
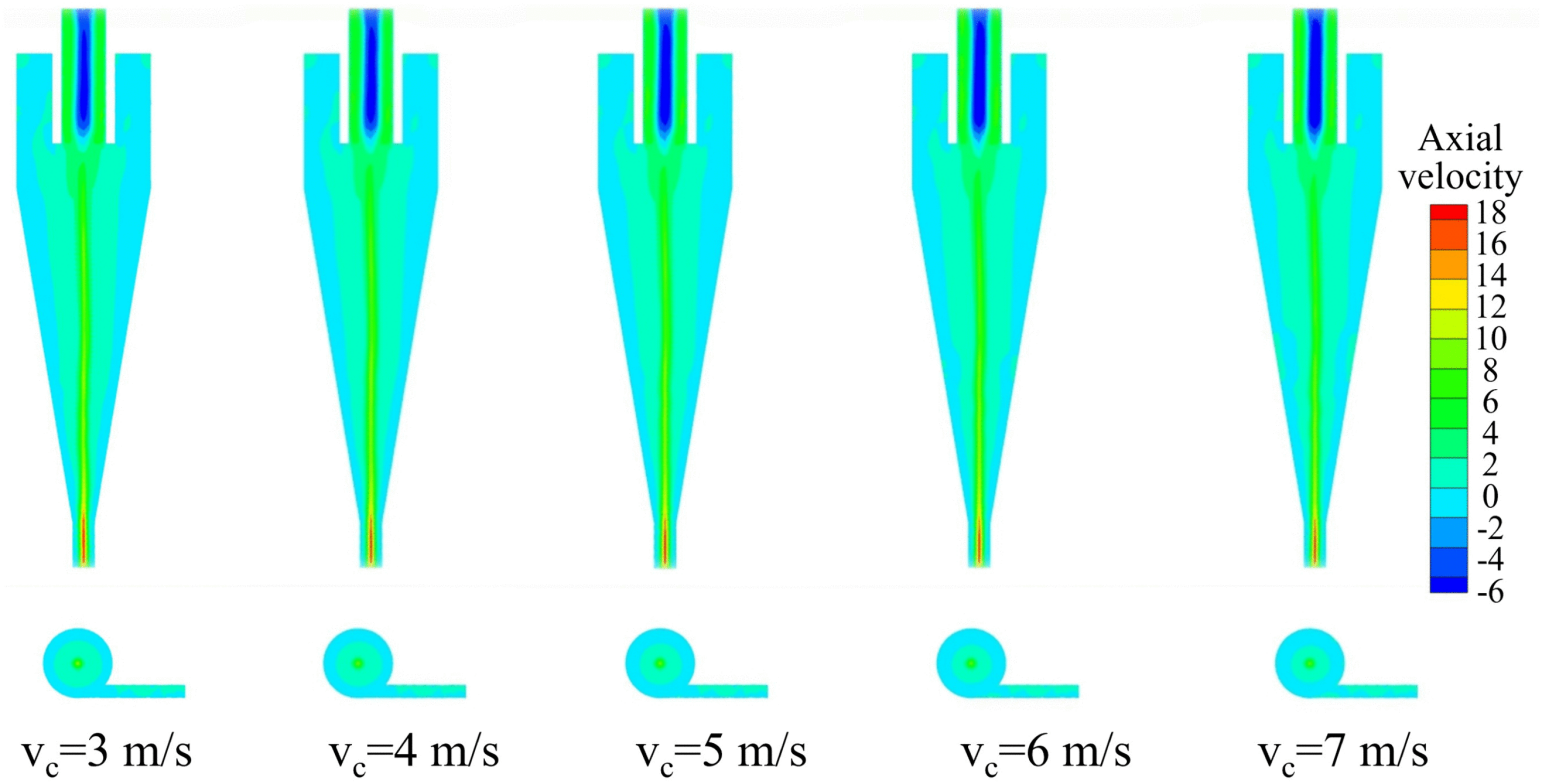
Axial velocity distribution under different impact velocities in the bottom-impact hydrocyclone.

**Fig 22:**
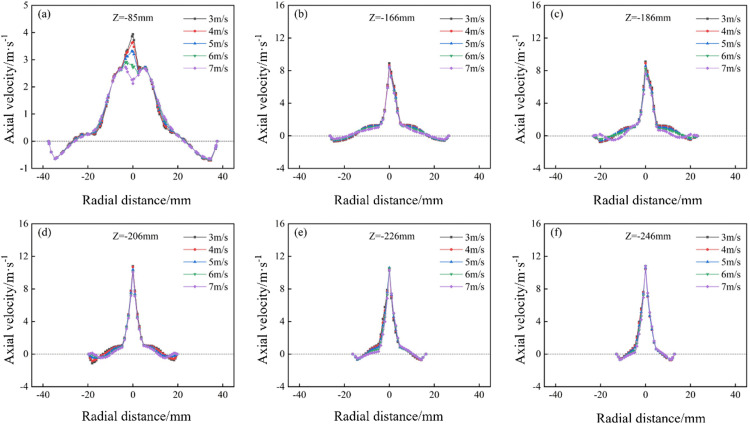
Axial velocity distribution at the X = 0 plane and different sectional heights under different impact velocities in the bottom-impact hydrocyclone. (a) Z = −85 mm, (b) Z = −166 mm, (c) Z = −186 mm, (d) Z = −206 mm, (e) Z = −226 mm, (f) Z = −246 mm.

The axial velocity distribution of the bottom‐impact hydrocyclone at different impact velocities is shown in Fig 21. The axial velocity distributions along the Y = 0 plane at Z = −85, −166, −186, −206, −226, and −246 mm are shown in Fig 22. As shown in Fig 21, the axial velocity field remained largely symmetric, indicating a reasonably stable flow. The impact velocity significantly affected the shape of the LZVV: at 3, 4, and 5 m/s, the LZVV was regular, reflecting good flow‐field stability. However, as the velocity increased further, the LZVV began to oscillate and zero‐velocity zones appeared near the wall, signaling a destabilized separation zone and impaired classification performance. The LZVV also shifted inward, enlarging the outer vortex region and causing fine particles to escape via the underflow. Fig 22(c) shows pronounced axial velocity fluctuations at excessively high impact speeds, further undermining stability.

**Fig 23:**
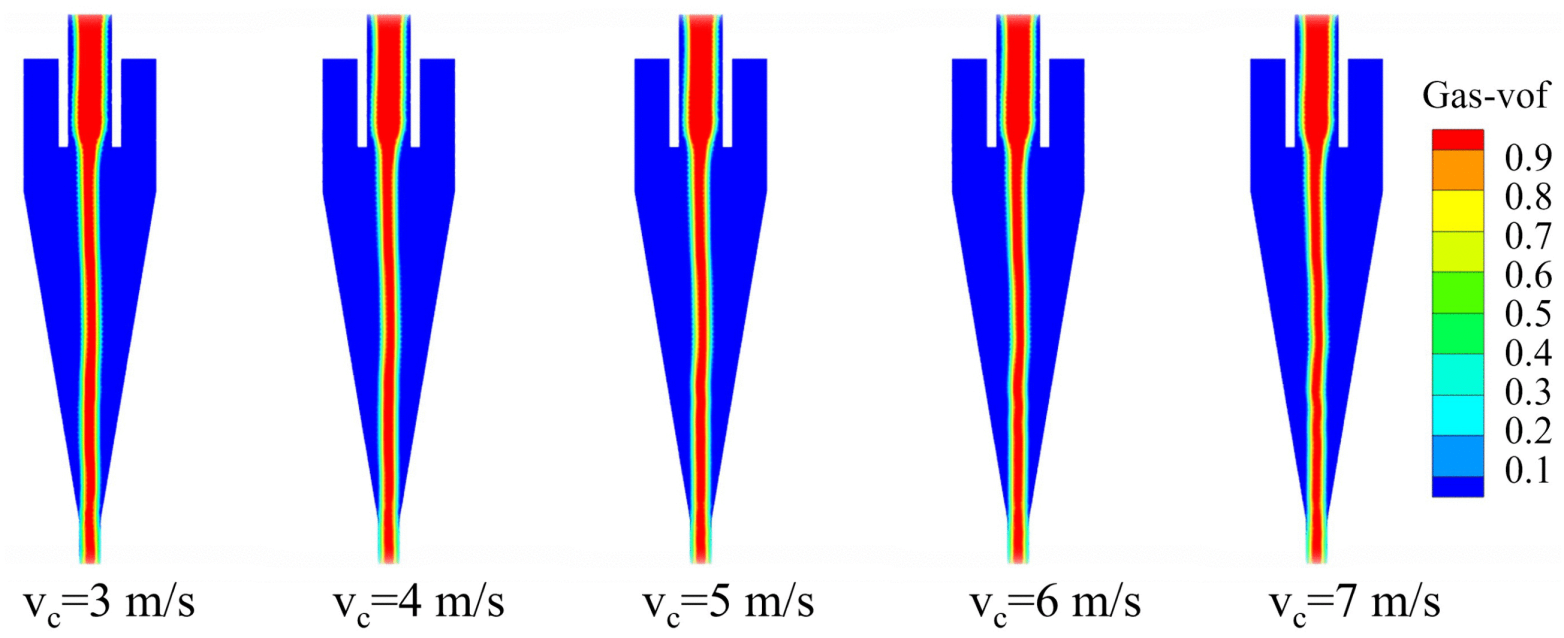
Air core cloud map of bottom-impact hydrocyclone at different impact velocities.

The air‐core distribution of the bottom‐impact hydrocyclone at different impact velocities is shown in Fig 23. As shown, at the velocities of 3, 4, and 5 m/s, the air core remained relatively stable, whereas at 6 and 7 m/s, slight oscillations appeared, which induced flow‐field instability.

To verify the reliability of the numerical simulation, the CFD-predicted internal flow-field characteristics were systematically compared with the experimental measurements. The simulation results show that the axial attenuation of tangential velocity, the typical “V-shaped” radial pressure distribution, and the evolution of the LZVV under different feed and impact velocities are all highly consistent with the experimentally observed variations in classification efficiency. Under higher impact velocities, the simulated tangential velocity and corresponding centrifugal force intensity are markedly enhanced, and the LZVV region becomes more compact and stable. The experiments exhibited the same trend, in which higher impact velocity led to a significant improvement in classification efficiency. In addition, the simulated evolution of the stable air-core region also agrees well with the experimental observations. These correspondences demonstrate that the CFD model can accurately capture the key flow-field features governing particle motion and separation performance within the hydrocyclone, thereby confirming the effectiveness and reliability of the numerical model in revealing the underlying separation mechanisms.

### 3.2 Construction and regulation of the precise hydrocyclone separation for Calcium and Magnesium components

#### 3.2.1. Experimental study on the conventional hydrocyclone.

**Fig 24:**
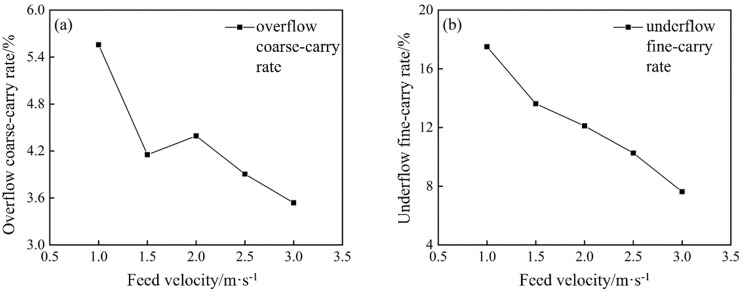
Overflow coarse‐carry rate and underflow fine-carry rate of the conventional hydrocyclone at different feed velocities.

In this experiment, the feed concentration was fixed at 12%, and inlet velocities of 1.0, 1.5, 2.0, 2.5, and 3.0 m/s were applied. Fig 24 shows the overflow coarse‐carry rate and underflow fine-carry rate of the conventional hydrocyclone at different inlet velocities.

As shown in Fig 24(a), the inlet velocity significantly impacts the overflow coarse‐carry rate. As the inlet velocity increased, the proportion of +0.074 mm particles in the overflow decreased, and the overflow coarse carryover rate decreased from 5.56% to 3.54%. This decline was primarily due to the stronger centrifugal field at higher velocities, which more effectively drove coarser particles toward the wall and into the underflow, thereby reducing the coarse carryover rate in the overflow. Although increasing the inlet velocity did lower the overflow coarse carryover rate, the overall reduction was modest.

As shown in Fig 24(b), the effect of inlet velocity on the underflow fine-carry rate was even more pronounced. With an increase in the inlet velocity, the proportion of −0.074 mm particles in the underflow decreased from 17.50% to 7.63%. This trend arose because the intensified flow field and centrifugal forces at higher velocities made it easier for coarser particles to escape via the underflow, thereby reducing the fraction of fine particles entrained. Additionally, the greater pressure differential at high velocities increased the centripetal buoyant force on the fine particles, enabling them to exit more readily through the overflow. Thus, higher inlet velocity was beneficial for minimizing fine‐particle entrainment in the underflow and improving the classification sharpness.

**Fig 25:**
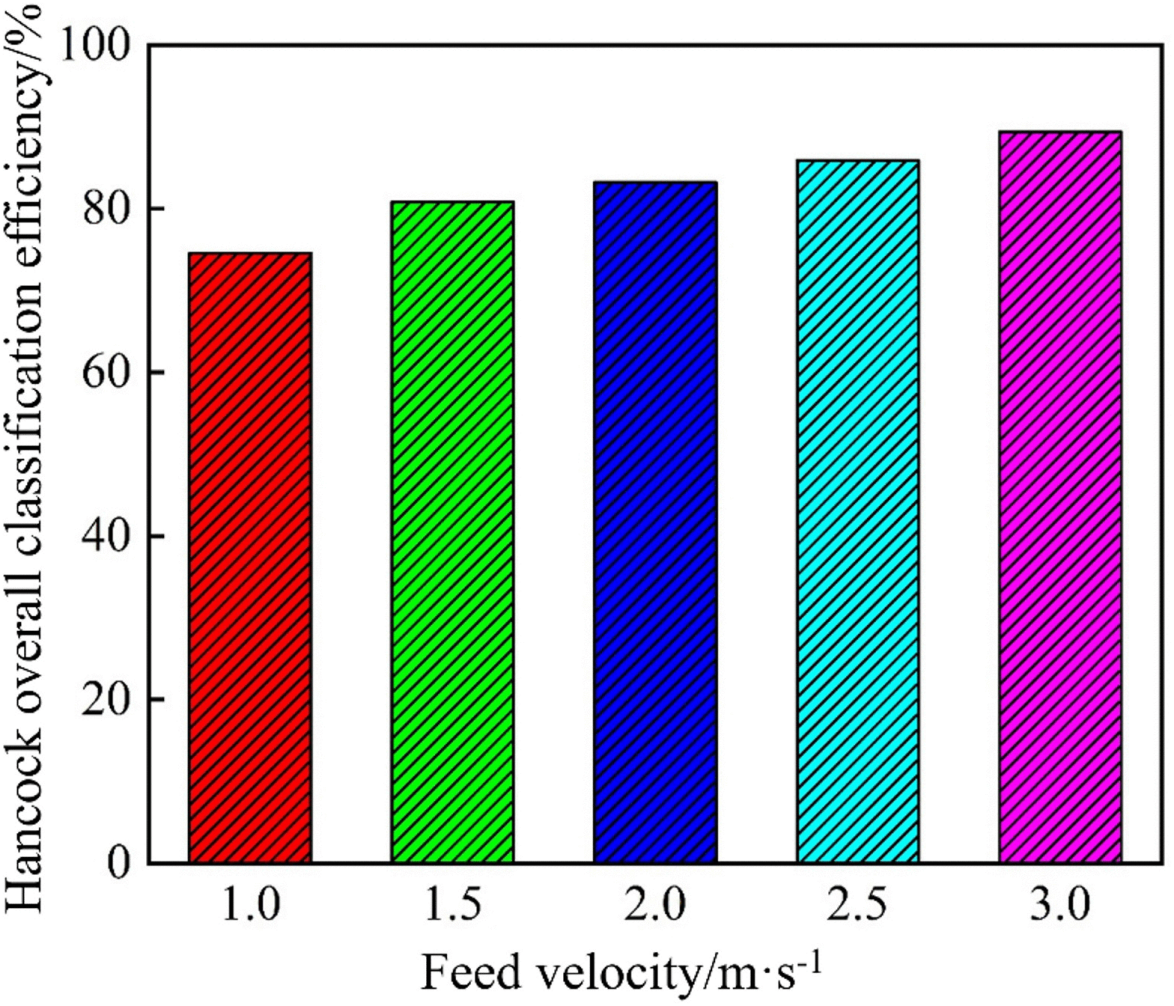
Hancock’s overall classification efficiency of the conventional hydrocyclone at different feed velocities.

Fig 25 shows the Hancock’s overall classification efficiency of the conventional hydrocyclone at different inlet velocities. As the inlet velocity increased, the Hancock’s classification efficiency steadily increased from 74.56% to 89.37%, representing an overall improvement of 14.81%. This increase was attributed to the stronger internal flow field and larger centrifugal forces at higher velocities, which amplified the force differential between coarse and fine particles and promoted more effective separation by both size and density, thereby significantly increasing the cyclone’s overall classification efficiency.

**Fig 26:**
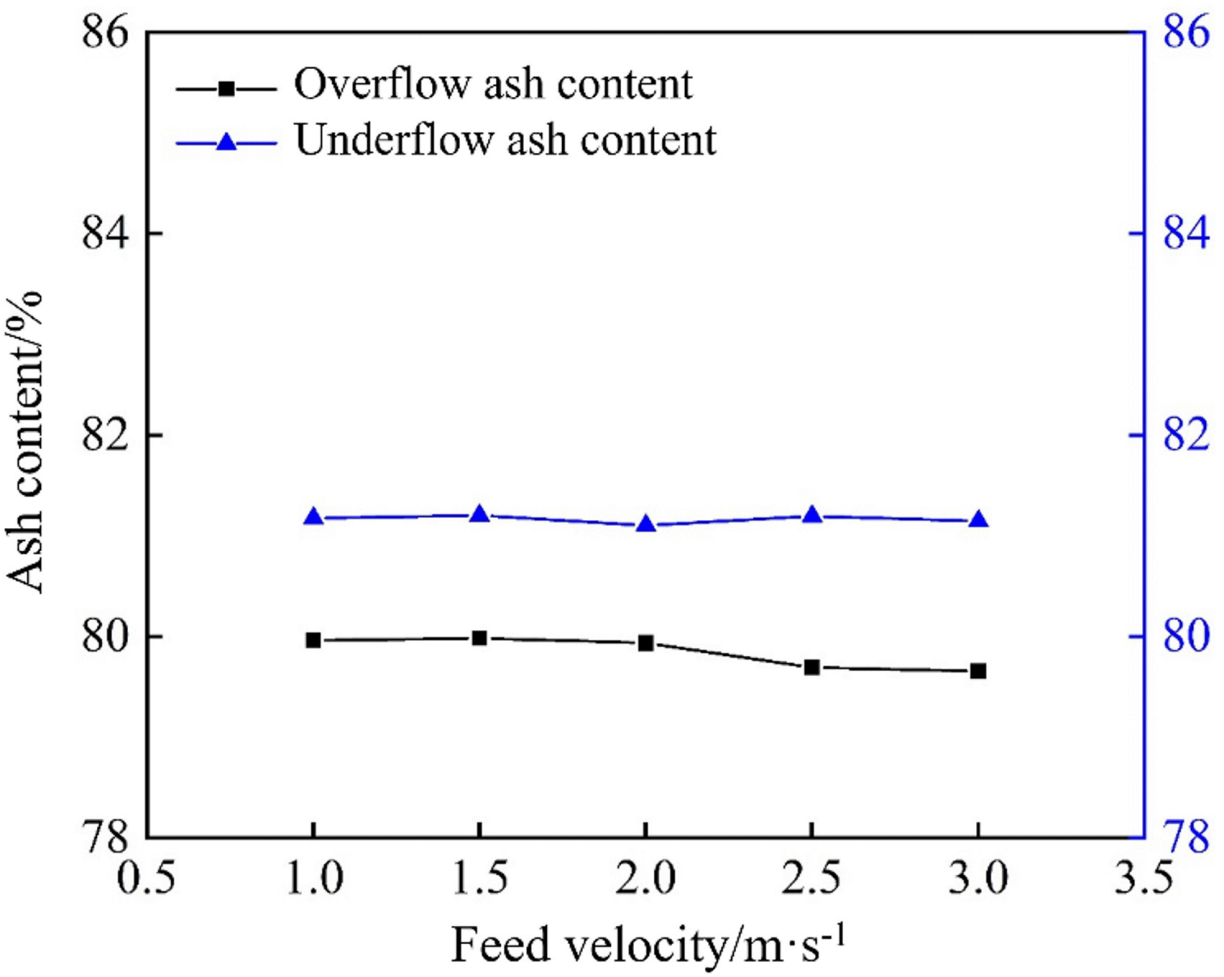
Ash content under different feed velocities in the conventional hydrocyclone.

The ash content is directly proportional to the density; higher ash content indicates higher density. To assess the effect of inlet velocity on density‐based separation in the conventional hydrocyclone, overflow and underflow samples were collected at each feed velocity, and their ash content was measured. As shown in Fig 26, the overflow ash content exhibited a slight downward trend with increasing velocity, although the change was minimal. The underflow ash content remained constant and was consistently higher than that of the overflow. This demonstrates that during separation, coal gangue with lower ash content and density mainly ends up in overflow, whereas gangue with higher ash content and density tends to settle in the underflow. Moreover, within the tested velocity range, variations in feed velocity had only a limited impact on ash content, indicating that the conventional hydrocyclone performed poorly in density‐based separation and that its structural or operational parameters must be optimized to improve the density separation efficiency.

**Fig 27:**
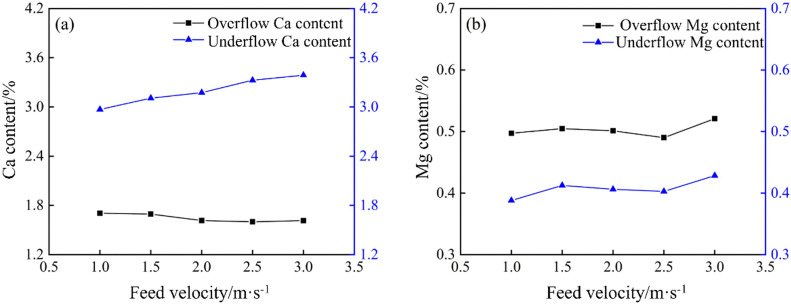
Calcium and magnesium content under different feed velocities in the conventional hydrocyclone. (a) Calcium content, (b) Magnesium content.

To investigate the effect of inlet velocity on the separation of calcium and magnesium components in the conventional hydrocyclone, overflow and underflow samples were collected at each feed velocity and analyzed by XRF. The results are shown in Fig 27.

As shown in Fig 27(a), the hydrocyclone exhibited a clear separation effect for calcium, and the calcium content in the underflow was consistently higher than that in the overflow, rising from 2.97% to 3.39% as the inlet velocity increased, whereas the overflow calcium content remained essentially constant with only minor fluctuations. This indicated that higher feed velocities significantly enhanced calcium enrichment. This may be because increasing velocity can strengthen the internal centrifugal forces, causing denser, coarser gangue particles, which preferentially host the calcium, to migrate more effectively into the underflow, thereby reducing fine particle entrainment.

As shown in Fig 27(b), magnesium separation was relatively weak. Although the overflow magnesium content was slightly higher than that of the underflow, they both remained nearly constant across the tested velocities: approximately 0.51% for the overflow and 0.41% for the underflow. This suggests that the inlet velocity has only a limited effect on magnesium separation, because the cyclone is primarily separated by particle size, and magnesium is distributed relatively uniformly across different size fractions.

#### 3.2.2. Experimental study on structural parameter optimization of bottom impact hydrocyclone.

In the previous section, the optimal feed velocity was determined. To enhance the ability of the conventional hydrocyclone to separate the calcium and magnesium components, a bottom-impact hydrocyclone was designed. Five variants, each with a differently sized impact pipe (L₅ = 40, 60, 80, 100, and 120 mm), were fabricated via 3D printing. The impact velocity was fixed at 5 m/s, and based on earlier feed velocity trials, the feed velocity was set to 3 m/s.

**Fig 28:**
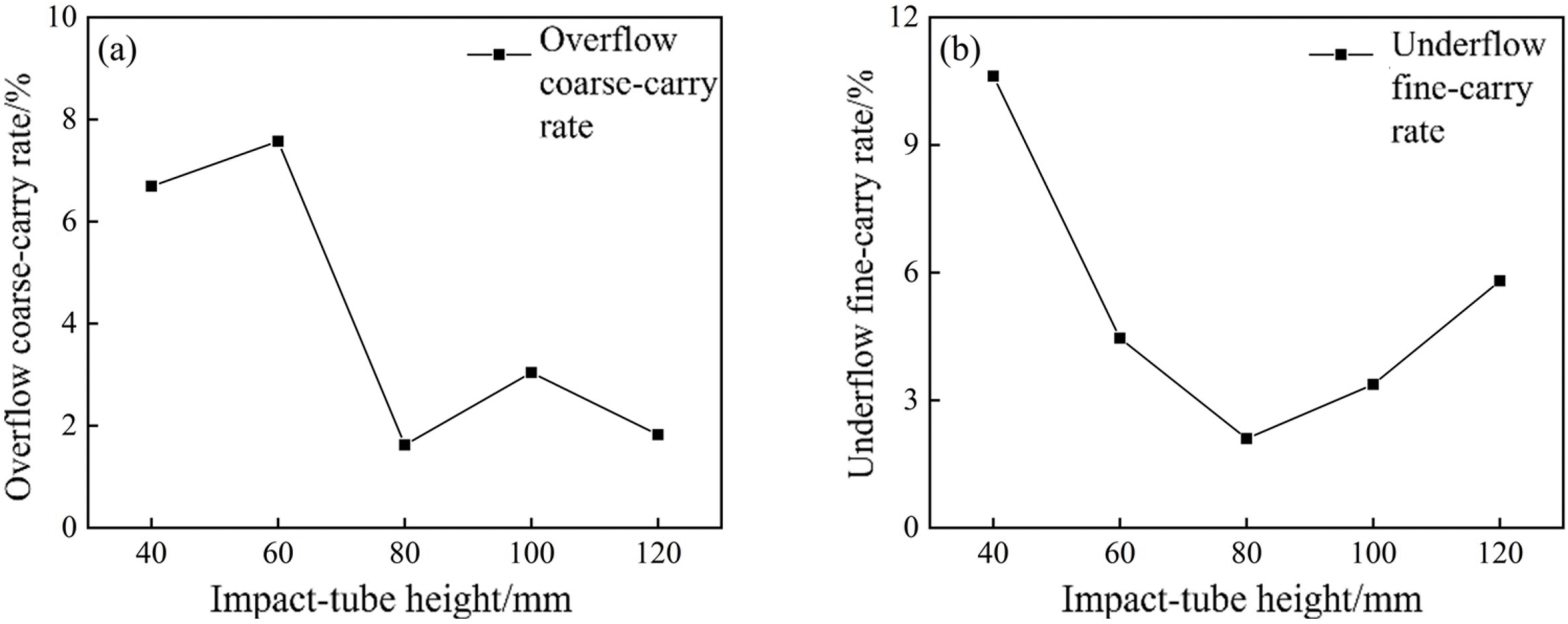
Overflow coarse‐carry rate and underflow fine-carry rate of the bottom-impact hydrocyclone at different impact pipe heights.

The overflow coarse‐carry rate and underflow fine-carry rate of the bottom-impact hydrocyclone at different impact pipe heights are shown in Fig 28.

From Fig 28(a), the overflow coarse‐carry rate was the highest at the lowest impact pipe height. As L₅ increased, the overflow coarse-carry rate steadily declined, reaching a minimum of 1.62% at 80 mm. This represents a reduction of 1.92% compared with the 3.54% coarse‐carry observed in the conventional cyclone, demonstrating that the introduction of the bottom‐impact stream substantially reduces coarse particle escape in the overflow.

Fig 28(b) shows that the underflow fine -carry rate (< 0.074 mm) initially decreased and then increased with rising L₅. Between 40 and 80 mm, the fine entrainment rate decreased monotonically, reaching a minimum of 2.10% at 80 mm. This indicated that a moderate impact pipe height effectively disrupted the sedimented particle bed at the cyclone base, reducing fine‐particle adhesion or agglomeration, and thus greatly improving size grading. However, at pipe height beyond 80 mm, the fine-carry rate increased, suggesting that an excessively tall impact pipe distorted the bottom flow structure, enlarged the underflow region, and allowed more fine particles to bypass the inner vortex. Compared with the 7.63% fine entrainment observed in the conventional cyclone, the optimized 80 mm configuration achieved a 5.53% reduction, confirming that careful selection of impact pipe height is critical for minimizing underflow entrainment and maximizing classification sharpness.

**Fig 29:**
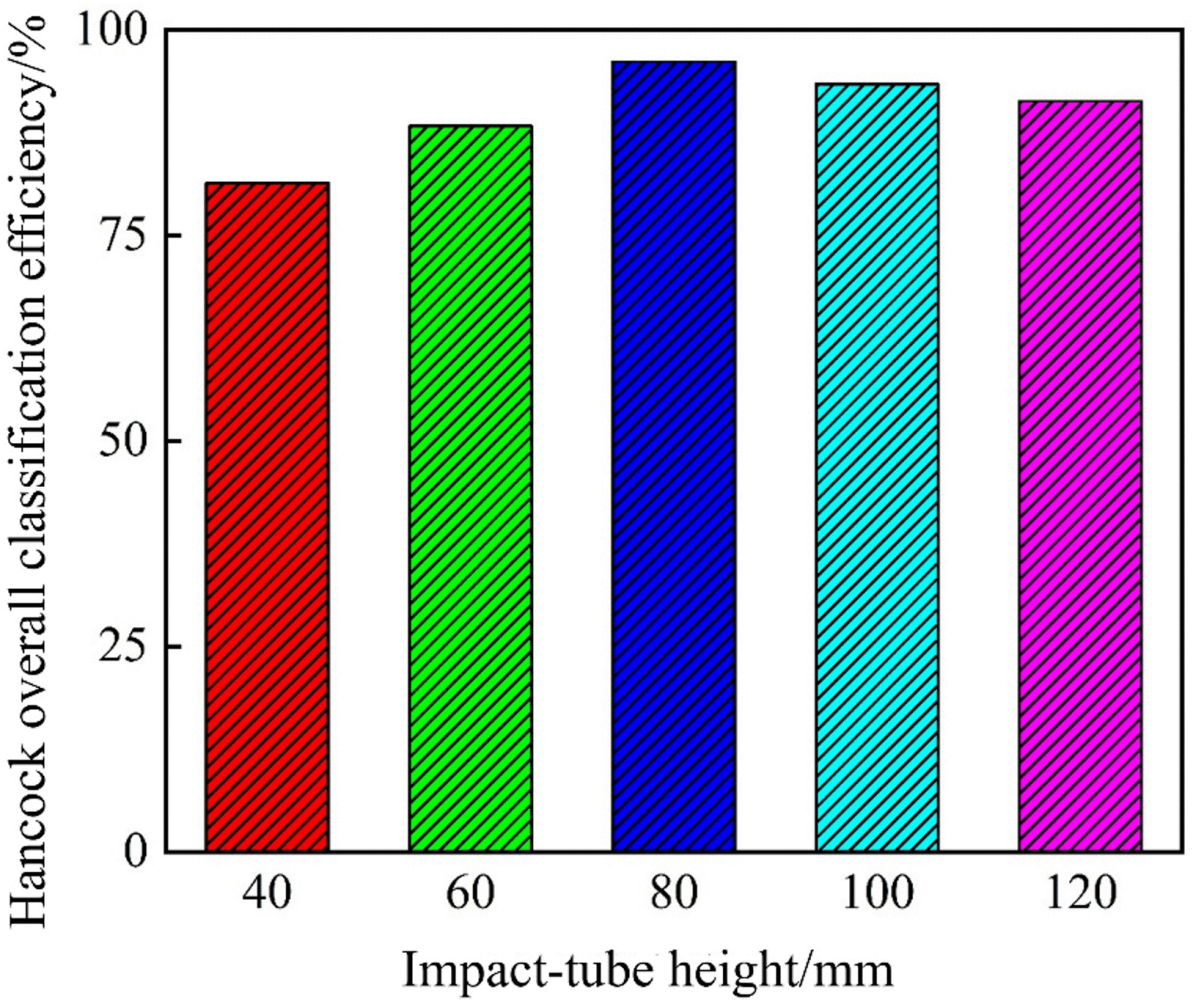
Hancock’s overall classification efficiency of the bottom-impact hydrocyclone at different impact pipe heights.

Fig 29 presents Hancock’s overall classification efficiency as a function of the impact pipe height. The efficiency curve rose from a low value at 40 mm, peaked at 80 mm, and then experienced a slight decline at heights of 100 mm and above. This behavior is similar to the trends observed in coarse carryover and fine entrainment. The 80 mm impact pipe provided the best balance between enhanced centrifugal separation and flow field stability, yielding the highest separation performance.

In summary, a comprehensive analysis showed that all key metrics were optimized at the impact pipe height of 80 mm. At this height, the overflow coarse‐carry rate reached its minimum of 1.62%, 1.92% improvement over the conventional cyclone, while the underflow fine coarse‐carry rate dropped to 2.10%, a 5.53% reduction. When the impact pipe height exceeded 80 mm, the fine entrainment rate increased and the overall classification efficiency declined slightly, likely due to alterations in the internal flow structure that impair particle grading. Therefore, setting the impact pipe height to 80 mm effectively maximized the classification performance of the cyclone.

**Fig 30:**
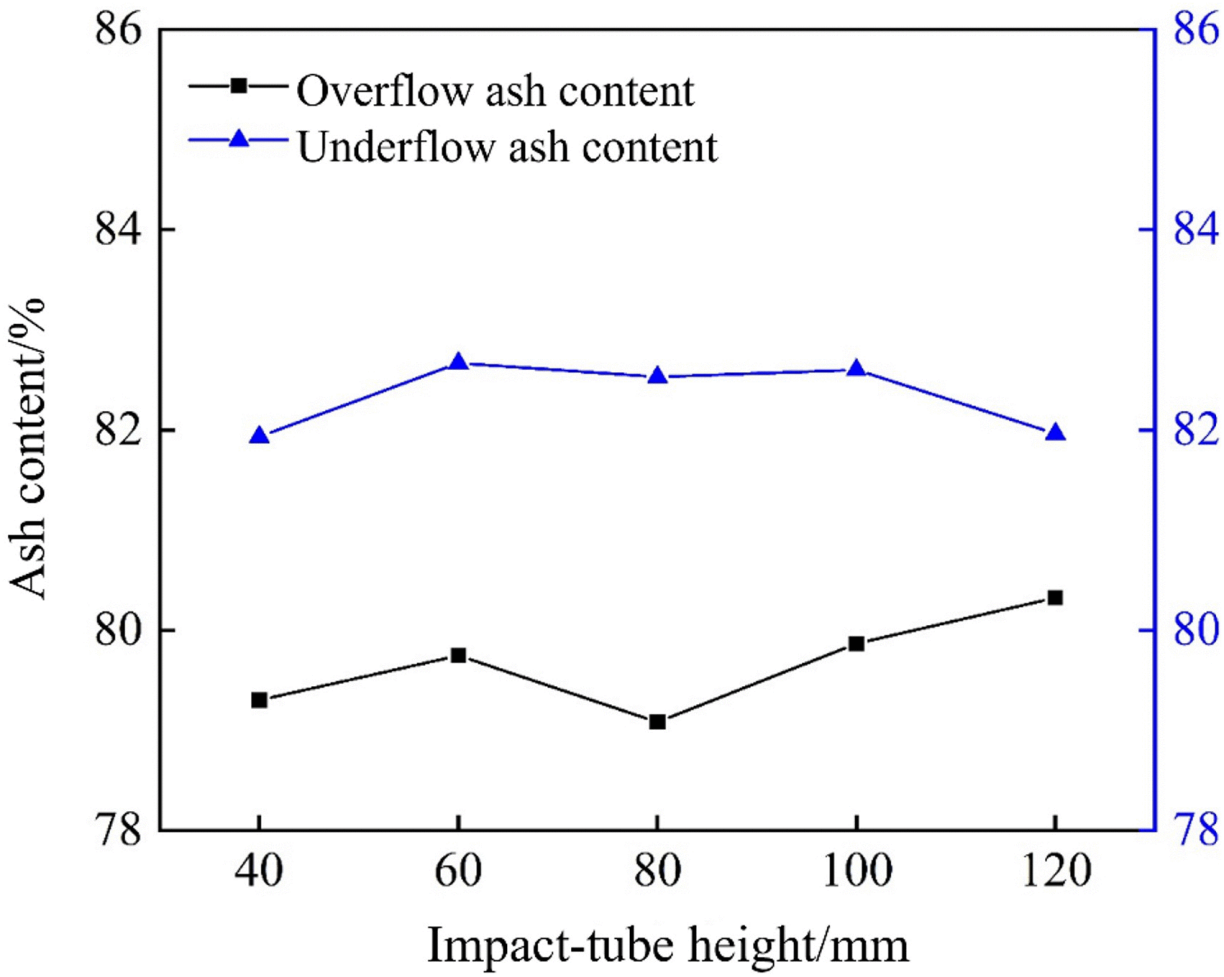
Ash content of the bottom-impact hydrocyclone at different impact pipe heights.

To study the impact of pipe height on density‐based separation, overflow and underflow samples were collected and analyzed for ash content. The results are shown in Fig 30. As shown, the overflow ash content fluctuated only slightly, between 79.5% and 80.5%, and reached its minimum at an L₅ of 80 mm; beyond this height, it rose modestly. This indicates that a moderate amount of impact water improves density separation, by facilitating better reporting of low‐ash (low‐density) gangue to the overflow, thereby improving separation sharpness. However, when L₅ exceeded 80 mm, excessive impact water disturbed the particle settling trajectories and weakened density‐based separation, causing the overflow ash to increase.

The underflow ash content was consistently higher, between 82% and 83%, and increased from L₅ = 40–60 mm before leveling off; at L₅ = 120 mm, it then declined. At low pipe heights, the impact stream was insufficient to fully loosen the sediment bed, allowing some low‐ash particles to report to the underflow. At moderate heights, the impact of water stabilizes the internal flow field and maximizes the density separation. However, at excessive heights, the altered flow structure permits more low‐ash particles to enter the underflow, thereby reducing its ash content.

**Fig 31:**
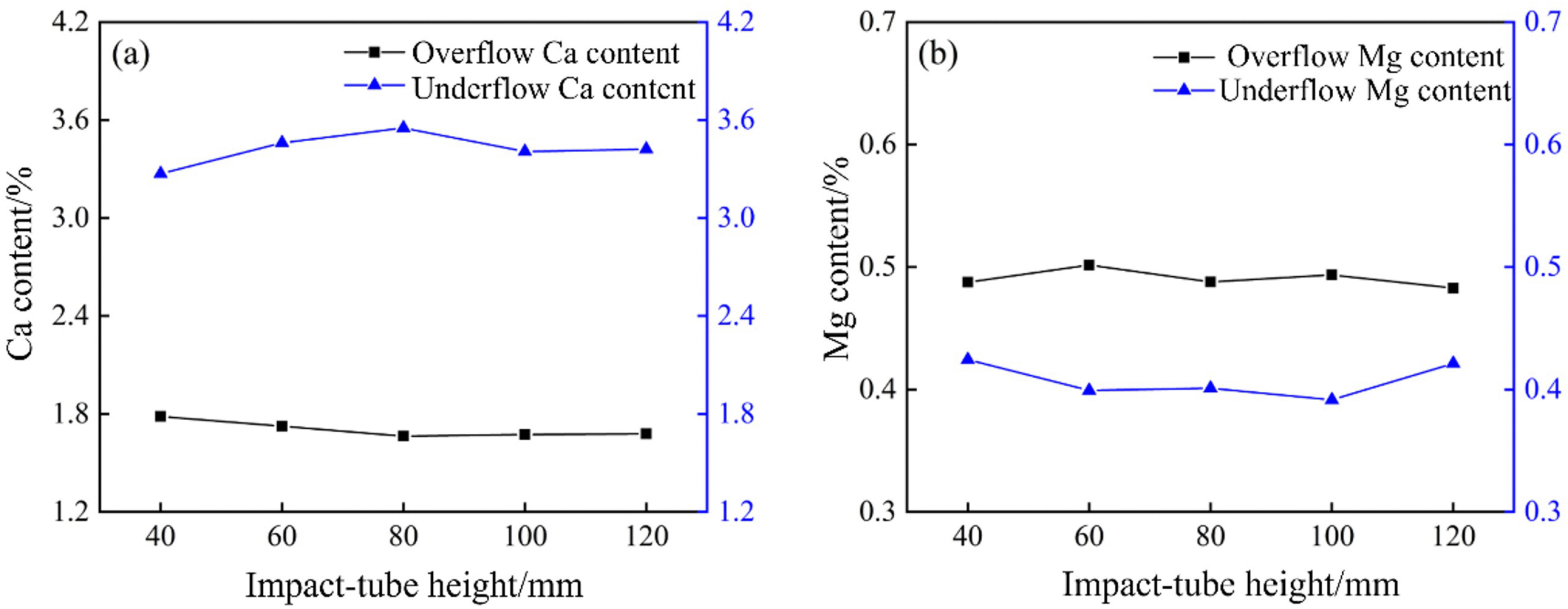
Calcium and magnesium content of the bottom-impact hydrocyclone at different impact pipe heights. (a) Calcium content, (b) Magnesium content.

XRF analysis was used to determine the Ca and Mg contents of overflow and underflow samples collected at different tube heights in the bottom-impact cyclone. As shown in Fig 31(a), the bottom‐impact cyclone can improve Ca enrichment, while impact‐tube height has a significant effect. Calcium was mostly concentrated in the underflow. As the tube height increased, the Ca content in the underflow increased and then stabilized, reaching a maximum of 3.53% at 80 mm, a 0.17% increase compared to the 3.39% measured in the conventional cyclone. The Ca content in the overflow decreased at first but then stabilized. These observations demonstrate that the introduction of the impact flow improves Ca separation, with optimal performance at an 80 mm tube height, in which both the overflow coarse-carry rate and the underflow fine‐carry rate are improved, resulting in an increased Hancock’s overall classification efficiency and a modest enhancement of density separation. Fig 31(b) shows the Mg separation performance of the bottom-impact cyclone, which is relatively weak, and the impact‐tube height has little effect on Mg enrichment. Overall, the Mg content in the overflow exceeded that in the underflow, fluctuating around 0.50% and 0.41%, respectively, indicating that the introduction of an impact flow can enhance the particle size classification, density separation, and Ca–Mg component separation in the cyclone, with the best results achieved at an impact‐tube height of 80 mm.

#### 3.2.3. Experimental study on operational parameter optimization of bottom impact hydrocyclone.

In the previous section, the structural optimization of the bottom-impact cyclone was performed. On this basis, the operational parameter and impact velocity were optimized to enhance classification, density separation, and Ca–Mg component separation. Based on studies of the feed velocity and impact-tube height, the feed velocity was fixed at 3 m/s, and the impact-tube height was 80 mm, while the impact velocity was varied at 3, 4, 5, 6, and 7 m/s.

**Fig 32:**
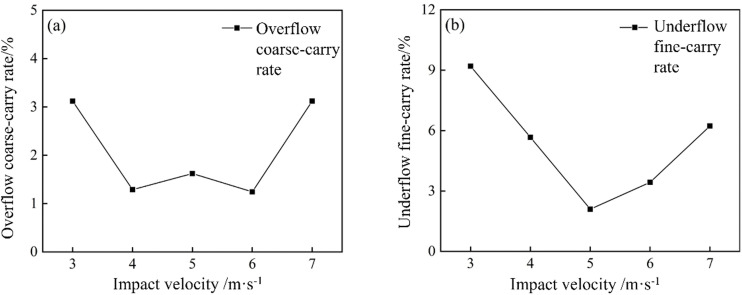
The overflow coarse‐carry rate and underflow fine-carry rate of the bottom-impact hydrocyclone at different impact velocities.

The overflow coarse‐carry rate and underflow fine-carry rate at different impact velocities in the bottom-impact cyclone are shown in Fig 32.

In Fig 32(a), the overflow coarse‐carry rate initially decreased and then increased as the impact velocity increased, reaching a minimum at approximately 5 m/s. At 3 m/s, the impact jet was too weak to fully dislodge the coarse particles at the cyclone base, allowing some to enter the overflow and resulting in a relatively high misplacement rate. As the impact velocity increased, the intensified jet drove more coarse particles into the underflow, reducing their carryover into the overflow and lowering the misplacement rate. However, beyond 5 m/s, the overly strong jet disrupted the flow field at the cyclone base, re-entraining coarse particles into the ascending flow and thus into the overflow, increasing the misplacement rate again. Therefore, an impact of velocity of approximately 5 m/s was most effective in minimizing coarse-particle misplacement into the overflow and improving the classification precision.

As shown in Fig 32(b), the underflow fine-carry rate also follows a “V-shaped” trend with the impact velocity, attaining its lowest value at 5 m/s. At low impact velocities, the jet was too weak, and the underflow slurry concentration remained high, which made it easier for fine particles to become entrained in the underflow, resulting in a high entrainment rate. As the velocity increased, the stronger jet more effectively dispersed the settled gangue particles at the base, reducing the fine-particle carryover and thus lowering the entrainment rate. However, when the velocity exceeded 5 m/s, the excessively turbulent base flow prevented some fine particles from entering the overflow and instead carried them into the underflow, causing the entrainment rate to rise again. Consequently, an impact velocity of 5 m/s most effectively minimized the fine-particle entrainment in the underflow and maximized the classification performance.

**Fig 33:**
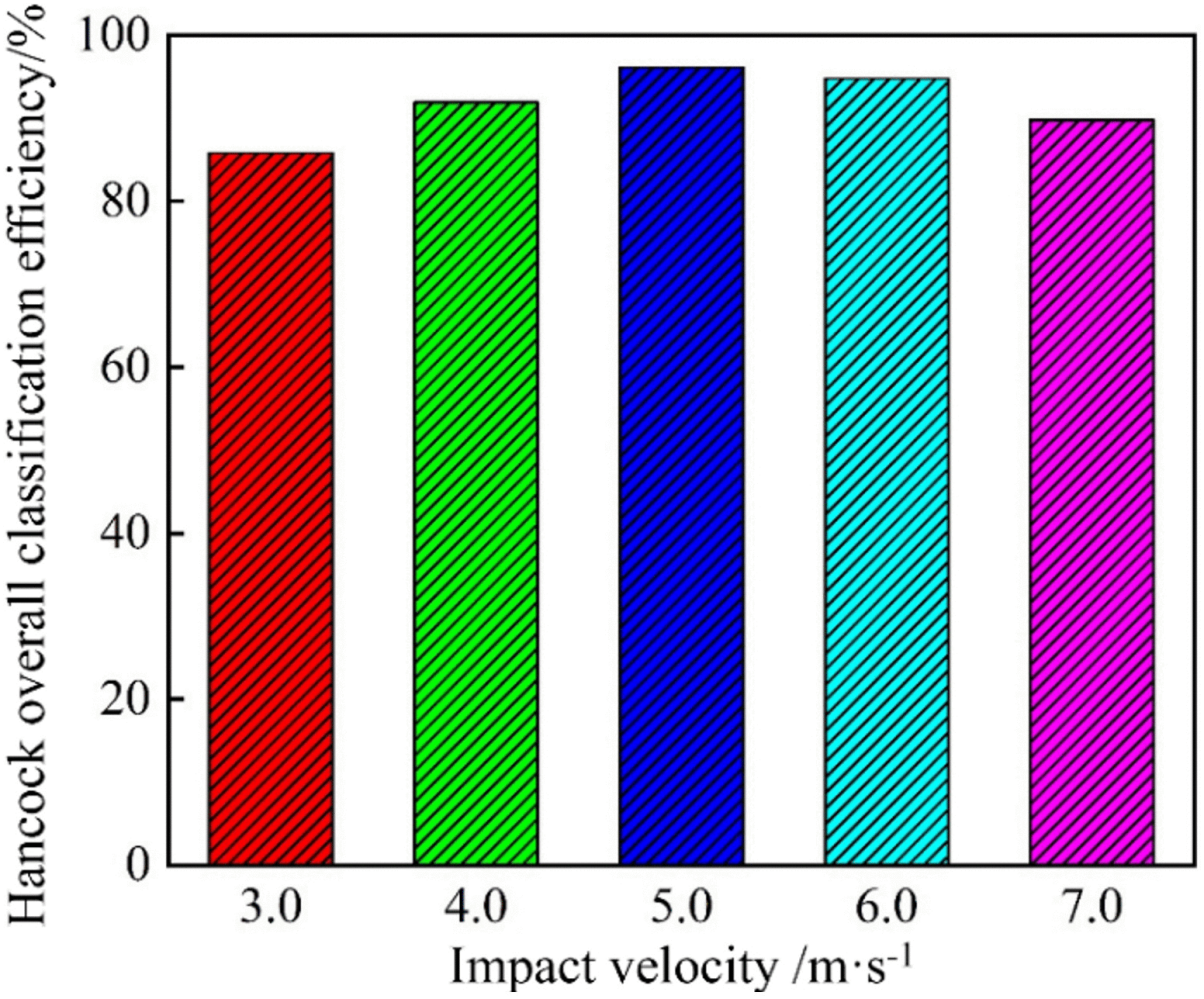
Hancock’s overall classification efficiency of the bottom-impact hydrocyclone at different impact velocities.

Fig 33 depicts the Hancock’s overall classification efficiency of the bottom-impact cyclone at different impact velocities. As the impact velocity increased, the efficiency first increased and then decreased, peaking at 96.13% at 5.0 m/s. At low impact velocities, the jet disturbance was too weak to fully loosen the material at the base of the cyclone, allowing fine particles to be carried into the underflow and coarse particles to misreport to the overflow, both of which reduced the efficiency. As the velocity increased to 5.0 m/s, the stronger jet effectively dispersed the settled gangue at the base, yielding the highest classification efficiency. However, beyond 5.0 m/s, excessive turbulence within the cyclone’s flow field disrupted particle settling—some fine particles failed to enter the overflow, and some coarse particles entered the wrong outlet—causing efficiency to decline. In summary, 5.0 m/s represents the optimal impact velocity, which maximizes particle settling while minimizing entrainment and misplacement; impact velocities that are either too low or too high impair classification performance.

**Fig 34:**
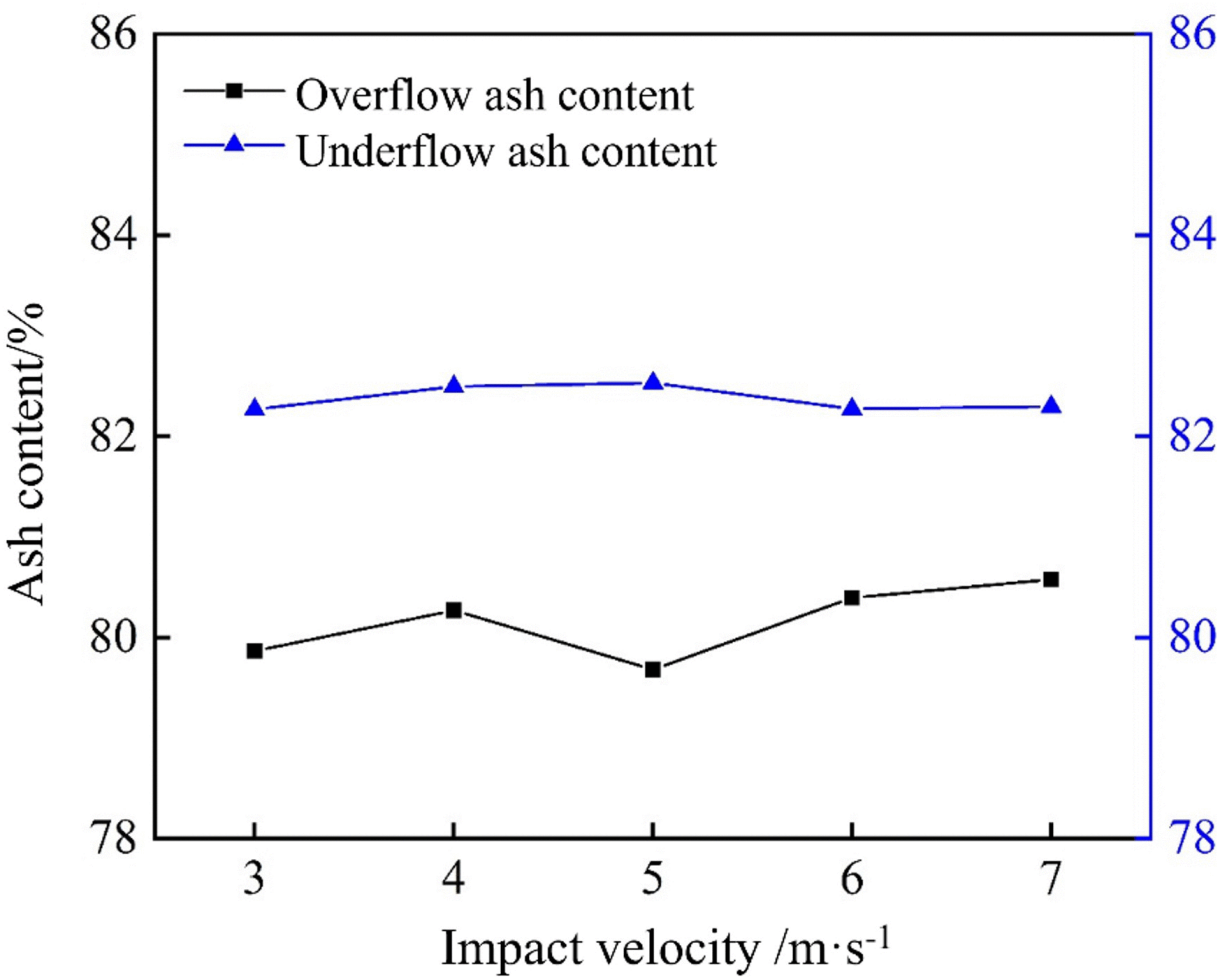
Ash content at different impact velocities in a bottom-impact hydrocyclone.

As shown in Fig 34, the underflow ash content was consistently higher than the overflow ash content, which aligned with the fundamental behavior of cyclone density separation. The underflow ash content initially increased and then decreased, peaking at an impact velocity of 5 m/s; however, the overall variation was small. This indicates that while impact velocity does influence density separation, its effect between 3 and 7 m/s is relatively minor. Similarly, the impact of velocity on overflow ash content was negligible across this range.

**Fig 35:**
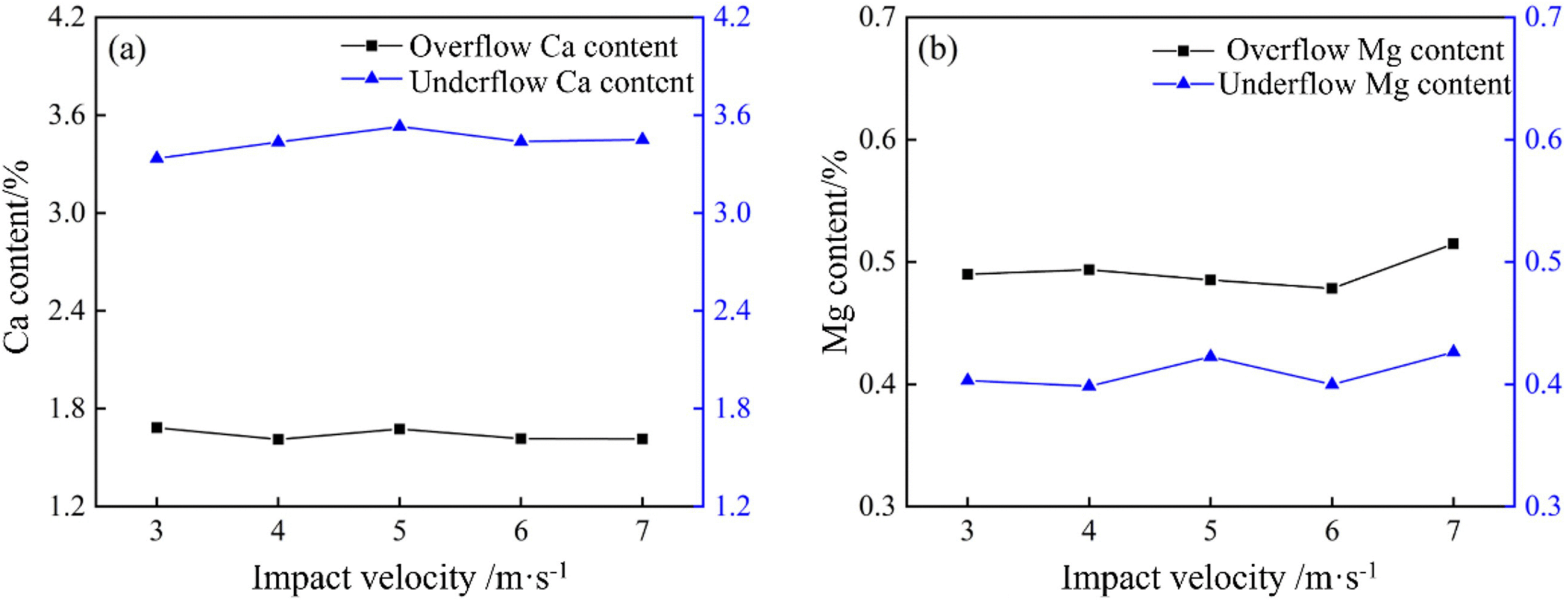
Calcium and magnesium content at different impact velocities in a bottom-impact hydrocyclone. (a) Calcium content, (b) Magnesium content.

The XRF results of the overflow and underflow samples are shown in Fig 35. Fig 35(a) shows how impact velocity affects Ca enrichment. The Ca content in the underflow initially increased and then decreased, peaking at 3.53% at 5 m/s. In contrast, the Ca content in the overflow remained essentially constant across the velocity range. This trend suggests that at 5 m/s, the reduced fine particle entrainment and improved classification sharpness maximize the separation of coarse, Ca-rich gangue particles into the underflow, thereby achieving the highest underflow Ca concentration.

As shown in Fig 35(b), the impact velocity had minimal influence on Mg separation. The Mg content in the overflow consistently exceeded that in the underflow, with average values of 0.50% and 0.41%, respectively. This is primarily because the cyclone operation is governed by the particle size classification, and Mg-bearing particles are distributed relatively uniformly across size fractions.

In summary, the bottom-impact hydrocyclone enhances internal pressure and tangential velocity by introducing an impact flow, thereby accelerating fluid motion within the flow field and generating more stable inner vortex. This effectively improves the stability of the LZVV and the air column, reinforces the centrifugal force field, and extends particle residence time through impact-induced reverse jets. These features make it particularly suitable for the separation and enrichment of calcium and magnesium components in coal gangue. Compared to conventional hydrocyclone, this design significantly improves enrichment efficiency. Moreover, as this optimization effectively enhances the centrifugal force field within the cyclone, thereby improving the separation efficiency of fine particles, the bottom-impact cyclone is also expected to deliver enhanced performance in areas such as the pre-concentration of fine-grained metal ores, plastic particle sorting, and electronic waste separation. This gives it broad potential for application and replication. At the same time, the improved structure remains relatively simple and has minimal impact on energy consumption, making it highly valuable for industrial applications. In addition, this device can serve as a direct replacement for the hydrocyclones currently used for coal classification, integrating well with existing processes without the need for significant additional equipment, which facilitates industrial application. However, the scaling-up criteria for the bottom-impact structure still require further investigation.

The high-velocity flow field and the reverse jet are the core features of the improved hydrocyclone, resulting in a notable enhancement in the size-based separation efficiency of coal gangue particles, a substantial reduction in the misplacement rate, and effective enrichment of calcium in the underflow. However, calcium and magnesium are primarily hosted in different minerals, and the magnesium-bearing phases exhibit much less variation in particle-size distribution. This behavior may be attributed to the generally finer particle size and more complex embedding of certain magnesium-containing minerals, leading to insufficient liberation and consequently weaker separation performance for Mg-bearing particles.

This makes it difficult to enrich magnesium through size-based separation, further deep separation followed by flotation or chemical leaching may be effective methods. Moreover, hydrocyclone generally perform poorly in density-based separation. Even though the introduction of the bottom impact flow enhances internal swirling and the centrifugal force field, particle size remains the dominant factor influencing separation efficiency—this is a fundamental limitation of hydrocyclone-based separation. As a result, the enrichment performance for magnesium in this study is notably lower than that for calcium.

## 4. Conclusion

In this study, coal gangue from the Wangjialing Coal Preparation Plant was used to investigate the multicomponent, microscale occurrence characteristics as well as the distribution characteristics of its Ca–Mg components. Using CFD, the structural and operational parameters of the conventional and the bottom-impact hydrocyclone were numerically investigated to elucidate the flow-field evolution and its effect on Ca–Mg component enrichment. Hydrocyclone separation experiments were then conducted to explore how these parameters influence Ca–Mg enrichment, yielding key process intensification techniques for fine-particle gangue separation. The principal findings were as follows:

(1) Raw coal gangue exhibited a high ash content of 81.66%, and calcium was uniformly distributed throughout, necessitating liberation by crushing before separation. After crushing to 0–1 mm, Ca became enriched in the coarser, higher-density fractions, allowing for size and density separation. Magnesium was primarily concentrated in the finer, higher-density fraction, although its overall distribution differences among fractions were minor.
(2) In the conventional cyclone, the internal static pressure was axially symmetric and decreased radially, dropping below zero at the center to form an air core. Higher feed velocities raised both wall pressure and tangential velocity, enhancing particle separation through stronger centrifugal forces. The axial velocity field comprised an outer and inner swirl, with the zero‐velocity envelope surface (LZVV) governing the separation performance. Feed velocity had little effect on the outer swirl. The air column fluctuated at the cyclone base, reaching its maximum diameter at the bottom of the overflow pipe, and grew with increasing feed velocity. In the bottom‐impact design, the impact‐tube height influenced flow‐field stability and separation performance. At 80 mm, both the pressure and tangential velocity increased, the LZVV symmetry improved, and the air core remained stable, all of which strengthened the centrifugal separation. Increasing the impact velocity further intensified the centrifugal field of the cyclone, but excessive velocity disrupted flow symmetry, amplified LZVV fluctuations, and destabilized the slurry flow, thereby impairing particle separation. The air core was most stable at an impact velocity of 5 m/s; beyond this velocity, slight oscillations appeared, which reduced the classification sharpness.
(3) At a feed velocity of 3.0 m/s in the conventional hydrocyclone, the Hancock classification efficiency reached 92.47%. When using the bottom-impact hydrocyclone (with an impact-tube height of 80 mm), the classification efficiency increased to 96.13% due to the bottom-impact effect, and the Ca content in the underflow rose to 3.53%, representing a 47.7% increase compared with the raw coal gangue. Although a certain degree of Mg enrichment was also observed, its extent was less pronounced compared with Ca. The Mg content in the underflow varied from 0.41% to 0.50%, corresponding to a moderate enrichment relative to the feed (0.46%). This behavior suggests that Mg-bearing particles participated in the separation process, but their enrichment was constrained by the finer particle size and more complex embedding of magnesium-containing minerals.
